# Metformin Alters Human Host Responses to *Mycobacterium tuberculosis* in Healthy Subjects

**DOI:** 10.1093/infdis/jiz064

**Published:** 2019-02-12

**Authors:** Ekta Lachmandas, Clare Eckold, Julia Böhme, Valerie A C M Koeken, Mardiana Binte Marzuki, Bastiaan Blok, Rob J W Arts, Jinmiao Chen, Karen W W Teng, Jacqueline Ratter, Elise J Smolders, Corina Van den Heuvel, Rinke Stienstra, Hazel M Dockrell, Evan Newell, Mihai G Netea, Amit Singhal, Jacqueline M Cliff, Reinout Van Crevel

**Affiliations:** 1 Department of Internal Medicine, Nijmegen; 2 Radboud Center for Infectious Diseases, Radboud University Medical Center, Nijmegen; 3 Nutrition, Metabolism and Genomics Group, Division of Human Nutrition, Wageningen University, Wageningen, the Netherlands; 4 Department of Immunology and Infection, Faculty of Infectious and Tropical Diseases, London School of Hygiene and Tropical Medicine, United Kingdom; 5 Singapore Immunology Network, Agency for Science, Technology, and Research; 6 Lee Kong Chian School of Medicine, Nanyang Technological University, Singapore; 7 Human Genomics Laboratory, Craiova University of Medicine and Pharmacy, Romania

**Keywords:** Metformin, tuberculosis, host-directed therapy, antimycobacterial mechanisms, gene transcription

## Abstract

**Background:**

Metformin, the most widely administered diabetes drug, has been proposed as a candidate adjunctive host-directed therapy for tuberculosis, but little is known about its effects on human host responses to *Mycobacterium tuberculosis*.

**Methods:**

We investigated in vitro and in vivo effects of metformin in humans.

**Results:**

Metformin added to peripheral blood mononuclear cells from healthy volunteers enhanced in vitro cellular metabolism while inhibiting the mammalian target of rapamycin targets p70S6K and 4EBP1, with decreased cytokine production and cellular proliferation and increased phagocytosis activity. Metformin administered to healthy human volunteers led to significant downregulation of genes involved in oxidative phosphorylation, mammalian target of rapamycin signaling, and type I interferon response pathways, particularly following stimulation with *M. tuberculosis*, and upregulation of genes involved in phagocytosis and reactive oxygen species production was increased. These in vivo effects were accompanied by a metformin-induced shift in myeloid cells from classical to nonclassical monocytes. At a functional level, metformin lowered ex vivo production of tumor necrosis factor α, interferon *γ*, and interleukin 1β but increased phagocytosis activity and reactive oxygen species production.

**Conclusion:**

Metformin has a range of potentially beneficial effects on cellular metabolism, immune function, and gene transcription involved in innate host responses to *M. tuberculosis.*

Diabetes increases susceptibility to tuberculosis [1] and worsens tuberculosis outcome [2]. The mechanisms behind this increase in susceptibility are unclear, and a role for diabetes drugs could be envisioned. In particular, the diabetes drug metformin has antiinflammatory effects and inhibits pathways such as mammalian target of rapamycin (mTOR) signaling, which are important in the host defense against *Mycobacterium tuberculosis* [3]. Nonetheless, metformin has been demonstrated to enhance mycobacterial clearance in mice [4] and is associated with lower rates of *M. tuberculosis* infection in humans [5]. Adding to that, the use of metformin in humans has been associated with a plethora of positive effects, potentially linked to glycemic control, such as a reduced risk of developing active tuberculosis [6, 7], a lower tuberculosis mortality rate [8], an increased tuberculosis treatment success rate, a reduced tuberculosis relapse rate [9], and enhanced culture conversion [9, 10].

Proposed mechanisms for metformin’s beneficial effects include an increase in mitochondrial reactive oxygen species (mROS) and enhanced killing of *M. tuberculosis*, but none of these have been investigated in humans. Importantly, the mechanism of action behind metformin’s effects are not clearly defined, because metformin acts through several pathways, including mitochondrial complex I inhibition; an increase in adenosine monophosphate (AMP)/adenosine triphosphate levels, leading to increased AMP activated kinase (AMPK) signaling; and decreased glucagon and mTOR signaling [11]. Last, it is challenging to study the effects of metformin in people living with diabetes, because characteristics of diabetes, such as hyperglycemia, dyslipidemia, vitamin D deficiency, and oxidative stress, may all affect immune responses to *M. tuberculosis* [12].

We therefore investigated the effects of metformin in humans without diabetes. We first characterized metformin’s effects on in vitro responses to *M. tuberculosis* and then validated these findings in vivo in healthy volunteers, showing that metformin alters mTOR signaling, inhibits p38 and AKT, rewires the blood cellular landscape, and enhances anti–*M. tuberculosis* responses.

## METHODS

### Healthy Volunteers and Functional Laboratory Assays

In the in vivo study, 11 healthy Dutch adults were given metformin in increasing doses, ending with a commonly used dose of 1000 mg twice daily. For all other in vitro experiments, blood specimens from healthy Dutch adults (estimated tuberculosis incidence, 1.5 cases/100 000) were subject to analysis in the presence or absence of metformin. Isolated peripheral blood mononuclear cells (PBMCs), CD14^+^ monocytes, or M1/M2 macrophages were stimulated with *M. tuberculosis* lysate for production of tumor necrosis factor α (TNF-α), interleukin 1β (IL-1β), interleukin 6 (IL-6), interleukin 10 (IL-10), interleukin 17A (IL-17A), interleukin 22, and interferon γ (IFN-γ). Proliferation of CD4^+^ T cells was measured by flow cytometry of carboxyfluorescein succinimidyl ester–labeled PBMCs stimulated for 6 days with *M. tuberculosis* lysate. Metabolic measurements included lactate production in stored cell culture supernatants, the redox ratio of nicotinamide adenine dinucleotide (oxidized; NAD^+^)/nicotinamide adenine dinucleotide (reduced; NADH) levels in cell lysates, glucose consumption, and mitochondrial mass and membrane potential. Activation of downstream mTOR target signaling was assessed by Western blot of phosphorylated (p)-AMPK, p-p70 S6K, p-4EBP1, p-P38, and p-AKT. Production of reactive oxygen species (ROS) was determined after incubation of whole-blood specimens or PBMCs with zymosan or *M. tuberculosis* lysate, by measurement of chemiluminescence after the addition of luminol. Phagocytosis was measured in PBMCs, using pHrodo Green Zymosan Bioparticles Conjugate and flow cytometry. *M. tuberculosis* infection was measured in PBMCs incubated with *M. tuberculosis* (H37Rv) at a multiplicity of infection (MOI) of 5 for 3 hours, lysed, and cultured on Middlebrook 7H11. Cellular viability of PBMCs was assessed by flow cytometry of Annexin V–FITC and propidium iodide–stained PBMCs.

### Transcriptomics

RNA sequencing (RNAseq; GSE102678) analysis was performed on participants’ samples before and after metformin administration, directly on ex vivo whole-blood specimens, and on isolated PBMCs following incubation with *M. tuberculosis* lysate. Libraries were prepared using stranded preparation reagents from Illumina and sequenced on a NextSeq500, generating approximately 36 million–45 million 43-bp paired-end reads per sample. Sequence files were aligned to the human genome, and aligned reads were counted. Differentially expressed genes were determined using the R package DESeq2, and gene set analyses were performed to determine how metformin affected biological pathways in vivo and in the in vitro response to *M. tuberculosis*. Quantitative reverse transcription–polymerase chain reaction (qRT-PCR) analysis was performed to validate RNAseq and functional assay results.

### CyTOF Marker Labeling, Data Acquisition, and Analysis

PMA and ionomycin stimulated PBMCs were stained with heavy-metal isotope–labeled antibodies (Supplementary Table 1) [13], barcoded, and acquired on CyTOF 1 (Fluidigm). Samples were debarcoded using manual gating in FlowJo, and analysis of live CD14^+/-^CD16^+/-^ monocytes was performed using the t-distributed stochastic neighbor embedding (tSNE) dimension reduction and Phenograph-based clustering algorithm [14]. See the Supplementary Methods for details on mass cytometry and statistical analysis.

### Statistics

All values are expressed as means ± standard errors of the mean of individual samples. Unless otherwise specified, data analysis was performed using the paired *t* test or the Wilcoxon signed rank test in GraphPad Prism software (GraphPad).

### Study Approval

Written informed consent was received from participants prior to their inclusion in the study. Experiments were conducted according to the principles expressed in the Declaration of Helsinki. Ethical approval of studies performed in vitro (NL32357.091.10) and involving healthy volunteers (NL47793.091.14) was granted by the Arnhem-Nijmegen Ethical Committee. As validation, ethylenediaminetetraacetic acid–anticoagulated blood from 10 healthy young subjects who received metformin (500 mg on days 1 and 2, increasing to 1000 mg on days 3–8) was examined as part of a pharmacokinetic study (NL53534.091.15). The human RNAseq study was approved by the London School of Hygiene and Tropical Medicine Research Ethics Committee (11968).

## RESULTS

### Metformin Regulates Cellular Metabolism and Cytokine Production in Humans

**Figure 1.**
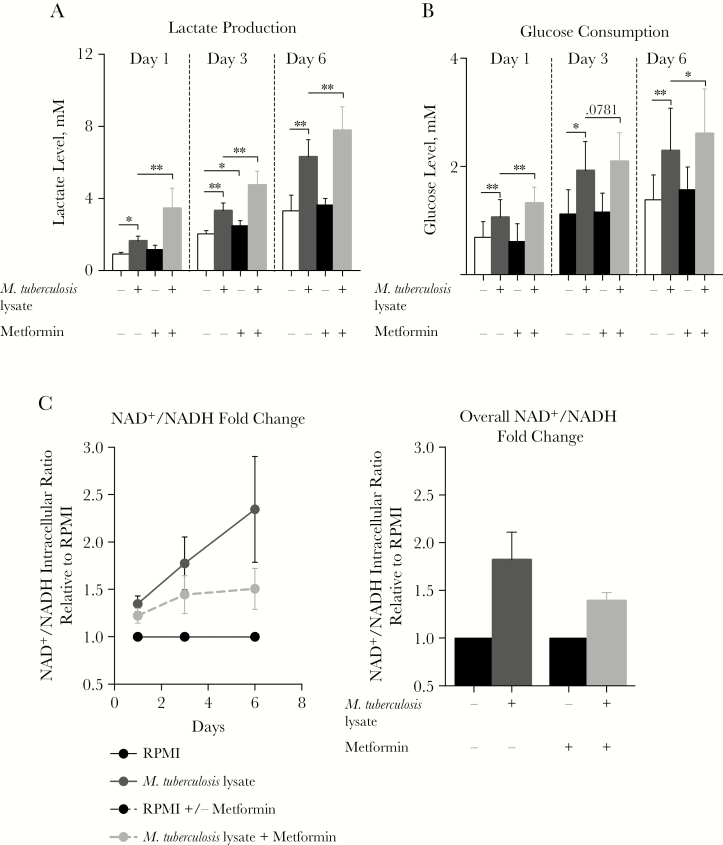
Metformin alters the mammalian target of rapamycin (mTOR) signaling axis while maintaining glucose regulatory effects. *A*, *C*, and *D*, Lactate production (*A*), glucose consumption (*C*), and fold change in nicotinamide adenine dinucleotide (oxidized; NAD^+^)/nicotinamide adenine dinucleotide (reduced; NADH) levels (*D*) in peripheral blood mononuclear cells (PBMCs) stimulated with *Mycobacterium tuberculosis* lysate in the presence or absence of 1000 µM metformin for 24 hours, 48 hours, or 7 days. For panel *A*, data are from 2 individual experiments. For panels *A*–*C*, data are shown as means ± standard errors of the mean from 2–3 experiments and 6–9 donors. RPMI, Roswell Park Memorial Institute medium. *P < .05 and ** *P* < .01, by the Wilcoxon matched-pairs signed rank test.

**Figure 2.**
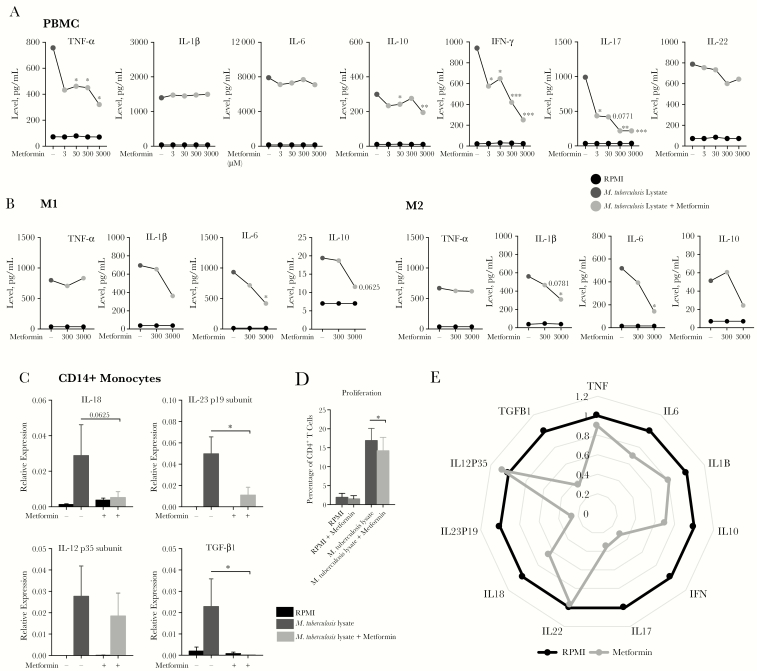
Metformin affects the cytokine profile of human cells stimulated with *Mycobacterium tuberculosis*. *A* and *B*, Cytokine production from human peripheral blood mononuclear cells (PBMCs; *A*) and monocyte-derived M1 and M2 macrophages (*B*) stimulated with *M. tuberculosis* lysate, with or without 3–3000µM of metformin for 24 hours (for tumor necrosis factor α [TNF-α], interleukin 6 [IL-6], interleukin 1β [IL-1β], and interleukin 10 [IL-10]) or 7 days (for interferon γ [IFN-*γ*], interleukin 17 [IL-17], and interleukin 22 [IL-22]). *C*, Cytokine gene expression in CD14^+^ monocytes stimulated with *M. tuberculosis* lysate with or without 3000 µM metformin after 4 hours (for interleukin 18 [IL-18] and transforming growth factor β1 [TGF-β1]) or 24 hours (for interleukin 23p19 [IL-23p19] and interleukin 12p35 [IL-12p35]). *D*, Percentage CD4^+^ T-cell proliferation in PBMCs stimulated with *M. tuberculosis* lysate in the presence or absence of 300 µM metformin for 6 days, using carboxyfluorescein succinimidyl ester labeling to track generations. *E*, Fold change in cytokine production by PBMCs stimulated with *M. tuberculosis* lysate with 3000 µM metformin, relative to stimulation in the absence of metformin. Values <1 indicate reduced cytokine production. This is indicated by projection toward the center of the radius. All data are means ± standard errors of the mean. For panels *A*–*C* and *E*, data are from 3 experiments and 6–13 donors. For panel *D*, data are from 4 experiments and 7 donors. *P < .05 and **P < .01, by the Wilcoxon matched-pairs signed rank test (for panels *A–C*) and the paired *t* test (for panel *D*).

We assessed the effects of metformin on glycolytic metabolism in human cells. When added to *M. tuberculosis* lysate–stimulated PBMCs from healthy individuals, metformin increased lactate production and glucose consumption (Figures 1A and 1B) while decreasing the ratio of the NAD^+^ level to the NADH level (Figure 1C). At both therapeutic concentrations (ie, 10–220 µM) and experimental concentrations [15], metformin showed clear effects on cytokine production. Depending on cell type, different concentrations of metformin significantly decreased *M. tuberculosis* lysate–induced production of (1) TNF-α, IL-10, IFN-*γ*, and IL-17 by PBMCs (Figure 2A); (2) IL-1β, IL-6, and IL-10 by M1 and M2 monocyte-derived macrophages (Figure 2B); and (3) TNF-α, IL-1β, and IL-10 by CD14^+^ monocytes (Supplementary Figure 1*A*). At a transcriptional level, metformin inhibited expression of *IL18*, *IL23P19*, and *TGFB1* (Figure 2C). The minimal effect of metformin on cellular proliferation (Figure 2D) is unlikely to account for the strong effects on cytokine production (Figure 2E). Finally, although findings were only suggestive rather than statistically significant, metformin also decreased the phosphorylation levels of the downstream mTOR targets p-p70S6K and p-4EBP1, while increasing phosphorylation of its known molecular target, AMPK (Supplementary Figure 1*B*). Metformin at the doses tested also had no significant effect on cellular viability (Supplementary Figure 1*C*).

### Transcriptional Profiling Reveals a Metformin-Related Gene Expression Signatures in Humans

**Figure 3.**
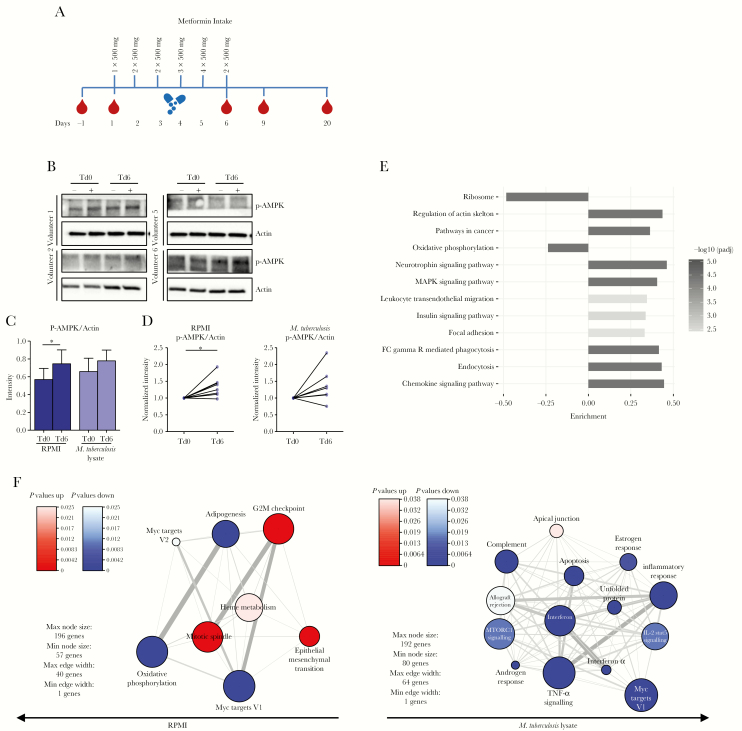
Global effects of metformin in healthy human volunteers. *A*, Healthy volunteers (n = 11) received an increasing dose of metformin for 5 consecutive days. Blood specimens were collected twice before and several times after metformin treatment. *B*, Western blot analysis of p-AMPK in lysates of peripheral blood mononuclear cells (PBMCs) collected from healthy volunteers before and after metformin intake and stimulated for 2 hours with Roswell Park Memorial Institute medium (RPMI; −) or *Mycobacterium tuberculosis* lysate (+). Four representative donors are shown. *C*, Quantitative relative band intensity analysis of phospho-AMPK (p-AMPK) before (treatment day 0 [Td0]) and after (Td6) metformin treatment in samples stimulated with RPMI or *M. tuberculosis* lysate. Data are means ± standard errors of the mean for 8 donors. *D*, Fold change in p-AMPK levels before (Td0) and after (Td6) metformin treatment in samples stimulated with RPMI or *M. tuberculosis* lysate. Data for 8 donors are shown. *P < .05 and **P < .01, by the paired *t* test. Western blot data are normalized to the loading control, actin. *E*, Gene set analysis of RNA sequencing data, showing Kyoto Encyclopedia of Genes and Genomes pathways that were differentially expressed in ex vivo blood samples following metformin administration. The bar length indicates the magnitude of the change of expression of the gene set. Data were analyzed using the Piano R package, and pathways with adjusted *P* values < .01 are shown. *F*, Hallmark gene set enrichment and network analysis, showing gene sets upregulated (red) or downregulated (blue) following metformin administration in resting PBMCs or those stimulated with *M. tuberculosis* lysate for 4 hours. The color intensity indicates the adjusted *P* value for the gene set enrichment. IL-2, interleukin 2; TNF-α, tumor necrosis factor α.

Next, we investigated the in vivo effect of metformin. Healthy subjects received standard dose of metformin, and blood specimens were collected at several time points before and after metformin intake (Figure 3A). As expected, the level of p-AMPK was increased in both unstimulated and *M. tuberculosis* lysate–stimulated PBMCs after metformin intake (Figure 3B–D and Supplementary Figure 2*A*). RNAseq analysis of whole-blood specimens revealed that metformin intake had no significant effects on individual genes (Supplementary Figure 2*B*). Instead, a consistent metformin-mediated effect was observed on combined sets of genes (Figure 3E), including significant downregulation of OXPHOS and ribosome pathways and significant upregulation of endocytosis/phagocytosis, MAPK, and chemokine signaling pathways.

In PBMCs, metformin intake led to differential expression of approximately 800 genes, both in unstimulated and *M. tuberculosis* lysate–stimulated cells (Supplementary Figure 2*C*). In unstimulated PBMCs, metformin intake led to upregulation of genes involved in mitosis and downregulation of genes involved in OXPHOS, adipogenesis and myc targets (Figure 3F). In *M. tuberculosis*–stimulated PBMCs, metformin intake led to suppression of genes involved in signaling of cytokines such as IFN-α, IFN-γ, and TNF-α, as well as genes involved in the OXPHOS and mTOR pathways (Figure 3F), all in line with the in vitro effects of metformin (Figures 1 and 2, respectively).

### Cytokine Responses to *M. tuberculosis* Are Suppressed by Metformin In Vivo

**Figure 4.**
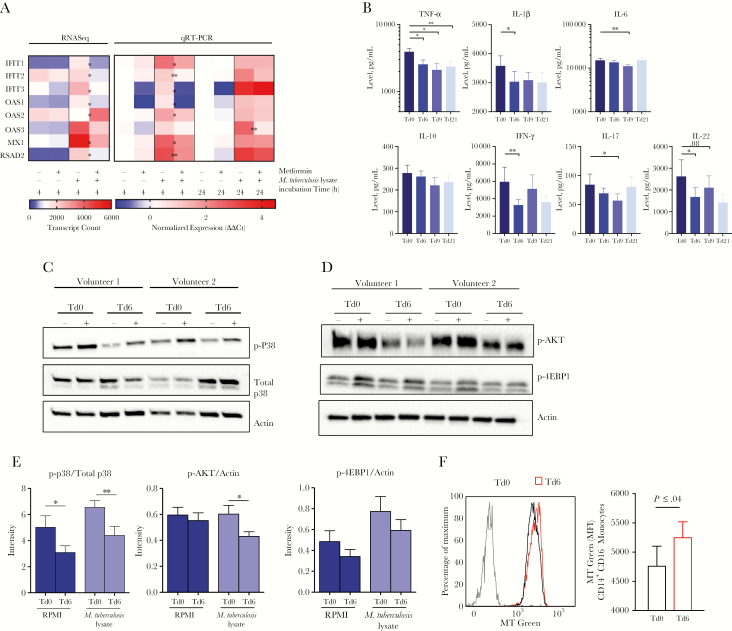
Metformin intake in healthy volunteers affects cytokine production via p38 and AKT inhibition. *A*, Expression of 8 genes in the “response to type 1 interferon” Gene Ontology group among peripheral blood mononuclear cells (PBMCs) collected before and after in vivo metformin administration from healthy volunteers and stimulated with *Mycobacterium tuberculosis* lysate in vitro for 4 or 24 hours. Expression measured by RNA sequence (RNAseq) analysis (at 4 hours) and quantitative reverse transcription–polymerase chain reaction (qRT-PCR) analysis (at 4 and 24 hours). *B*, Cytokine production from isolated PBMCs collected before and after metformin intake and stimulated with *M. tuberculosis* lysate for 24 hours (for tumor necrosis factor α [TNF-α], interleukin 6 [IL-6], interleukin 1β [IL-1β], and interleukin 10 [IL-10]) or after 7 days (for interferon γ [IFN-*γ*], interleukin 17 [IL-17], or interleukin 22 [IL-22]) in the presence of 10% pooled human serum. *C* and *D*, Findings of Western blot analysis of phospho-p38 (p-p38) and total p38 levels (*C*) and p-AKT and p-4EBP1 levels (*D*) in lysates of PBMCs collected from healthy volunteers before and after metformin intake and stimulated for 2 hours in Roswell Park Memorial Institute medium (RPMI; −) or *M. tuberculosis* lysate (+). Data are representative of 4 of 8 evaluated donors from the trial. Western blot data are normalized to the loading control, actin. *E*, Fold change in p-p38/total p38 levels, p-AKT/actin levels, or p-4EBP1/actin levels in PBMCs collected before (treatment day 0 [Td0]) versus those collected after (Td6) metformin treatment and stimulated with RPMI or *M. tuberculosis* lysate. *F*, Mitochondrial mass assessment in CD14^+^CD16^-^ monocytes. The left panel is overlay of data from before and after metformin treatment in a specimen from the same individual. The right panel should the median fluorescence intensity (MFI) yielded by MitoTracker Green (MT) in 3 samples. Gray, FMO control. *P < .05 and **P < .01, by the paired *t* test. Western blot data are mean values ± standard errors of the mean and are representative of 8 donors presented in panels *C* or *D* or Supplementary Figure 3*A* or 3*B*.

Each gene ontology (GO) group in the identified gene sets was investigated, and the “response to type 1 interferon” GO set showed the most markedly reduced expression in ex vivo *M. tuberculosis* lysate–stimulated PBMCs from individuals taking metformin (Supplementary Figure 2*D*). Within this GO set, the expression of 8 genes (*IFIT1*, *IFIT2*, and *IFIT3*, which encode IFN-induced protein with tetratricopeptide repeats 1, 2, and 3, respectively; *OAS1*, *OAS2*, and *OAS3*, which encode 2′-5′-oligoadenylate synthase 1, 2, and 3, respectively; *MX1*, which encodes MX dynamin-like GTPase 1; and *RSAD2*, which encodes radical S-adenosyl methionine domain containing 2) was reduced >2-fold following metformin administration in cells stimulated with *M. tuberculosis* lysate for 4 hours (Figure 4A) and, to a lesser extent, after stimulation for 24 hours, as shown by qRT-PCR analysis (Figure 4A). Additionally, metformin intake led to a significant decrease in TNF-α, IL-1β, IL-6, IFN-*γ*, and IL-17 release in response to *M. tuberculosis* lysate (Figure 4B), with effects on cytokine production up to 21 days after metformin intake. Collectively, our results indicate that metformin inhibits the *M. tuberculosis*–induced type 1 IFN response and inflammation in human PBMCs.

### Metformin Regulates the AKT-mTOR Pathway and Mitochondrial Metabolism in Humans

Because the MAPK, AKT, and mTOR pathways are known to strongly influence cytokine production, levels of p-P38 and total P38 (Figure 4C and Supplementary Figure 3*A*) and p-AKT and p-4EBP1 (Figure 4D and Supplementary Figure 3*B*) were measured in PBMCs before and after metformin intake. An overall decrease in the phosphorylation of all 3 targets was observed. Quantitative band intensity analysis showed that the ratio of the p-P38 level to the total P38 level, the level of p-AKT/actin, and the level of p-4EBP1/actin were in most cases significantly reduced because of metformin intake (Figure 4E). Supplementary Figure 3*C*–*E* demonstrate the effects on phosphorylation at an individual level. For further evidence, we analyzed the effect of metformin on the gene expression levels of these enzymes and found decreased expression of *AKT2* (fitting with metformin’s role in homeostasis) and increased expression of *PRKAB2* (which encodes a regulatory subunit of AMPK; Supplementary Figure 3*F*). Because AKT and mTOR are central metabolic regulators [16, 17], we investigated the effects of metformin on mitochondrial mass (Supplementary Figure 4*A*). Metformin increased the mitochondrial mass of CD14^+^CD16^-^ classical monocytes, as demonstrated by an increased Mitotracker green median fluorescence intensity (Figure 4F). This increase was not observed for CD14^-^CD16^+^ nonclassical monocytes (Supplementary Figure 4*B*). This highlights metformin-mediated alterations in mitochondrial functionality in CD14^+^CD16^-^ classical monocytes, which may correlate with the antiinflammatory effect of metformin [18, 19].

### Metformin Modulates the Peripheral Monocyte Landscape in Humans

**Figure 5.**
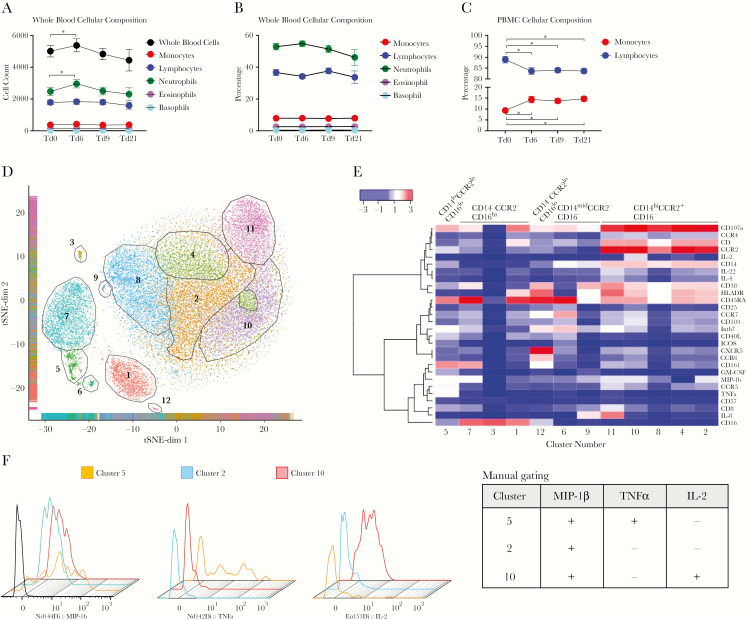
Metformin intake in healthy volunteers alters the blood cellular composition landscape. *A*–*C*, Analysis of leukocyte counts plotted as raw cell counts for whole blood (*A*), as percentage of total counts for whole blood (*B*), and as percentage of total counts for isolated peripheral blood mononuclear cells (PBMCs; *C*). *D*, Cryopreserved PBMCs collected before (treatment day 0 [Td0]) or after (Td6) metformin intake were stimulated with PMA-ionomycin and analyzed by mass cytometry. t-distributed stochastic neighbor embedding (tSNE) analysis of single-cell data from blood monocytes in analyzed samples. Cells were plotted and color-coded on the basis of the 12 “unsupervised” Phenograph clusters. *E*, Heat-plot summary of the average median expression of each marker analyzed for the 12 clusters identified. Twelve clusters are divided into 5 subsets, based on the expression of CD14, CD16, and CCR2. *F*, Mass cytometry data were analyzed by a manual gating strategy. The 3 differentially regulated monocyte clusters were overlayed to assess the expression of cytokines. The table on right indicates the depiction of (in terms of + [expression] and – [no expression]) which cluster expresses which cytokine, based on the manual gating strategy.

Metformin intake altered the number and distribution of circulating immune cells. In whole-blood specimens, metformin led to a transient increase in the total white blood cell (WBC) and neutrophil counts (Figure 5A) without altering the relative distribution of cell types (Figure 5B). In PBMCs, metformin increased the proportion of monocytes and decreased the proportion of lymphocytes (Figure 5C).

To achieve a single-cell systems-level perspective of the effect of metformin on monocytes, PBMCs from blood specimens collected before (ie, on treatment day 0 [Td0]) and after (ie, on Td6) metformin intake were stimulated with phorbol ester and ionomycin, stained with a panel of 38 surface and intracellular cytokine markers (Supplementary Table 1), and analyzed using CyTOF [20]. We first verified the panel antibodies for their binding to the PBMCs (Supplementary Figure 5) and then gated out the pure population of monocytes (CD3^-^CD19^-^CD56^-^γδTCR^-^Vd1^-^VD2^-^CD57^-^CD161^-^CD14^+/-^CD16^+/-^) for analysis (Supplementary Figure 6). Analysis of monocytes by using tSNE in conjunction with a Phenograph-based clustering algorithm [14, 21] identified 12 distinct cell clusters with shared surface and intracellular marker expression characteristics (Figures 5D and 5E). Based on the expression of CD14, CD16, and CCR2, the 12 clusters were divided into 5 monocyte subsets (Figure 5E), illustrating significant heterogeneity among the classical and nonclassical monocyte populations in humans. Three of 12 clusters were found to be significantly enriched or depleted in samples from Td6, compared with samples from Td0. These differentiated clusters included the following diverse activated phenotypes: CD14^hi^CD16^-^MIP-1β^+^IL-2^-^TNFα^-^ (cluster 2, downregulated), CD14^hi^CD16^-^MIP-1β^+^IL-2^+^TNFα^-^ (cluster 10, downregulated), and CD14^lo^CD16^lo^MIP-1β^+^IL-2^-^TNFα^+^ (cluster 5, upregulated; Figure 5F). The accuracy of machine-learning automated gating when validated by manual gating showed that clusters 2 and 10 were CD14^hi^CD16^-^ and that cluster 5 was CD14^lo^CD16^mid^ (Supplementary Figure 7*A*). Furthermore, manual gating revealed that only clusters 5 and 10 expressed TNF-α and IL-2, respectively (Figure 5F), similar to findings of tSNE analysis, but that all 3 clusters (ie, clusters 5, 10, and 2) expressed macrophage inflammatory protein 1β (Figure 5F), confirming the results of tSNE analysis. The manual gating strategy also indicated a trend toward decreased total population frequencies of CD14^hi^CD16^-^ classical monocytes or increased total population frequencies of CD14^-^CD16^+^ nonclassical monocytes (Supplementary Figure 7*B*). Collectively, our results delineate the effect of metformin on the functional capacity of heterogeneous peripheral monocytes.

### Metformin Enhances Innate Host Defense Pathways in Exposed Human Leukocytes

**Figure 6.**
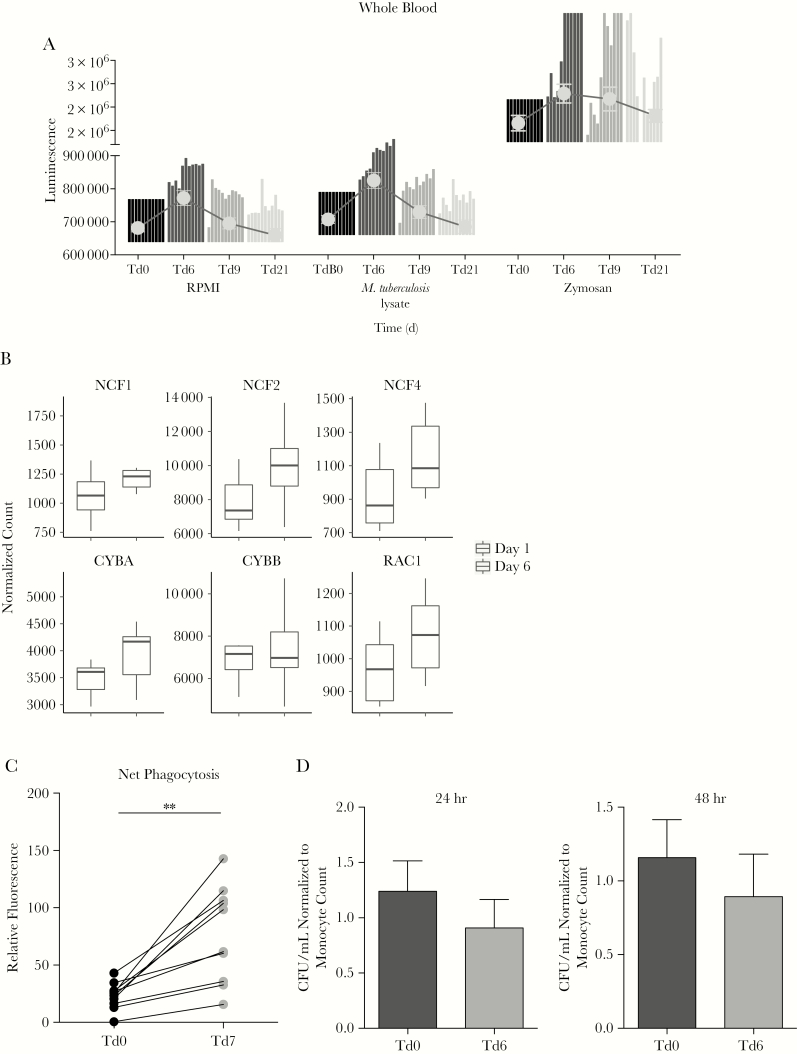
Metformin intake in healthy volunteers affects ex vivo antimycobacterial defense mechanisms but not *Mycobacterium tuberculosis* outgrowth. *A*, Reactive oxygen species (ROS) production, as measured by luminol reaction, in whole-blood specimens collected from volunteers before and after metformin treatment and stimulated with Roswell Park Memorial Institute medium (RPMI), *M. tuberculosis* lysate, or zymosan. Data are representative of 11 individual donors. Bars representing the fold change in production in specimens collected on treatment day 6 (Td6), Td9, or Td21 over that in specimens collected on Td0 for each individual donor are superimposed with gray dots representing mean values ± standard errors of the mean. *B*, Expression of 6 genes encoding key NADPH oxidase proteins for ROS production were assessed in ex vivo blood specimens by RNA sequencing analysis (RNAseq) before and after administration of metformin in the healthy volunteers. *P < .05 and **P < .01, by the Wilcoxon matched-pairs signed rank test. *C*, Net phagocytosis of pHrodo conjugates in healthy volunteers given metformin for 7 days. Upon RBC lysis, blood cells were incubated with the pHrodo suspension for 2 hours in an incubator without elevated CO_2_ levels at 37°C before measuring fluorescence. *D*, Numbers of colony-forming units (CFU) per milliliter after 24 hours or 48 hours of infection of peripheral blood mononuclear cells that were obtained from volunteers before and after metformin treatment and then infected with mycobacteria. Data were normalized to the monocyte count.

Metformin intake showed clear effects on innate host defense mechanisms. ROS production was strongly upregulated in whole-blood samples immediately after metformin treatment (Td6), both spontaneously and upon stimulation with *M. tuberculosis* lysate and zymosan (Figure 6A). In line with increased ROS production in whole-blood specimens, genes involved in ROS production, such as *CYBB* (which encodes NADPH oxidase 2), *CYBA* (which encodes p22-PHOX), *RAC1* (which encodes Rac family small GTPase 1), and, particularly for ROS production in neutrophils, *NCF1* (which encodes p47-PHOX), *NCF2* (which encodes p67-PHOX), and *NCF4* (which encodes p40-PHOX), were strongly upregulated in blood specimens after metformin intake (Figure 6B). The increase in ROS production did not correlate with an increase in white blood cell or neutrophil counts (Supplementary Figure 8*B* and 8*C*). No increase in ROS production was observed in isolated PBMCs (Supplementary Figure 8*A*).

RNAseq analysis of whole-blood specimens revealed that metformin upregulated genes involved in endocytosis, such as receptors (RTKs and GPCR), regulators of clathrin-mediated pit formation (*AP2*) and clathrin uncoating (Hsp70), and regulators of intracellular vesicular trafficking (Arfs, ArgGAPs, and ArfGEFs; Supplementary Figure 9*A*). Increased phagocytosis activity following metformin intake was confirmed in a second group of healthy subjects taking metformin, using zymosan-labeled beads in whole-blood specimens (Figure 6C). The increase in phagocytosis activity correlated with an increase in white blood cell counts but not neutrophil counts (Supplementary Figure 9*B*). Furthermore, in vitro metformin-pretreated PBMCs also showed upregulated phagocytosis (Supplementary Figure 9*C*). Finally, we examined the effect of metformin on the killing of *M. tuberculosis*. Of 8 subjects, metformin led to restricted ex vivo growth of *M. tuberculosis* in 4 subjects. Overall, there were no significant differences (Figure 6D). The colony-forming unit counts were unaffected by normalization to monocyte numbers.

## DISCUSSION

A study in mice and retrospective human data suggest that metformin, the most widely used diabetes drug, may improve the outcome of tuberculosis [4, 6, 8]. We examined how metformin modulates the peripheral immune cell distribution, its gene expression, and its functional output in humans, using high-dimensional phenotypic and RNA analyses. Metformin administration was found to dampen proinflammatory cytokine production while promoting phagocytosis and ROS production, possibly through the generation of nonclassical monocytes, which are implicated in trained innate immunity [22]. These functional changes were associated with an inhibition of the type 1 IFN pathway, a decrease in p-AKT and p-P38 signaling, and an increase in AMPK signaling. Our data are in line with increasing evidence that metformin possesses antiinflammatory properties, considered to be mediated in part via alterations in cellular metabolism [23].

A strong effect of metformin on inflammatory cytokine signaling was observed both in vitro and in vivo. Metformin inhibited the type I IFN response by blocking the expression of the IFN-stimulated genes *IFIT1*, *IFIT2*, and *IFIT3*, which among other activities, regulate inflammatory cytokine messenger RNA stability, cell proliferation, and apoptosis [24]. Neutrophil-driven type 1 IFN signaling in blood, including upregulated *IFIT1*, *IFIT2*, *IFIT3*, and genes similar to those in our data [25], but not type 1 IFNs themselves have been identified as a signature of active tuberculosis [26], and inhibiting this pathway by using zileuton, an arachidonic acid metabolism modulator, protects mice from tuberculosis [27]. Our data show that metformin can downregulate the type 1 interferon pathway in humans.

ROS production and phagocytosis activity were increased by metformin, and this was not explained by altered cell counts, suggesting that the observed effects are intrinsically mediated by metformin. This is supported by the accompanying transcriptional changes observed in both ROS and phagocytosis-related genes and the increase in phagocytosis activity induced by metformin in vitro. Mechanistically, AMPK activation has been linked to phagocytosis activity as pharmacologic [28, 29] or genetic [30, 31] ablation of AMPK subunits negatively influenced phagocytosis. It would be interesting to investigate the effect of metformin on autophagy in future studies and to compare this effect to findings from an elegant study showing that autophagic capacity does not correlate with *M. tuberculosis* susceptibility in mice [32]. As ours is the first exploratory study of the effects of metformin on host defense in vivo in nondiabetic individuals, future studies should examine the effect of metformin on the phagocytic capacity of specific cell types, such as macrophages and dendritic cells.

Metformin intake increased ex vivo mycobacterial killing capacity of PBMCs in some individuals but not all. In earlier work, we found that mycobacterial survival decreased in metformin-treated human macrophages [4]. This effect of metformin was reversed by the inclusion of ROS-scavenging agents. It is possible that 5 days of metformin exposure in vivo is too short, that the effect of metformin on killing capacity of PBMCs is somewhat lost during cryopreservation, or that other cells such as neutrophils contribute to the antimycobacterial effects of metformin. Future studies could use bronchiolar lavage cells to investigate control mechanisms from the disease site, rather than in peripheral blood. Alternatively, metformin could have subtle effects on mycobacterial killing and bigger effects on ameliorating inflammation. Whereas proinflammatory cytokines are required for the control of *M. tuberculosis*, the balance between proinflammatory and antiinflammatory cytokines is important for the restriction of mycobacterial growth and prevention of overt pathology [33, 34]. Here, we found that metformin dampens the expression of proinflammatory cytokines while enhancing antimycobacterial processes such as phagocytosis and ROS production.

In mice, we have previously shown metformin-mediated restriction of *M. tuberculosis* outgrowth [4], although another study found no additive effect of metformin when combined to the standard tuberculosis treatment [35]. In diabetic patients with tuberculosis, metformin use has been linked with more-rapid culture conversion [9], particularly in patients with cavitary lung disease and a high bacterial burden [10], and with better treatment outcomes [9], indicating that the net result of all the effects of metformin is enhanced mycobacterial control in vivo. In a cohort of 296 diabetic patients with tuberculosis in Singapore [4], metformin was associated with lower mortality, and a similar association was found in a cohort of 634 diabetic patients in Taiwan [8]. However, neither cohort study included microbiological data. The survival difference could equally be explained by the well-known beneficial effects of metformin on cardiovascular mortality or by its immunomodulating effects, as found in this study. Future clinical trials in nondiabetic patients with tuberculosis will help establish the effect of metformin on clinical and microbiological outcomes of tuberculosis treatment.

Metformin is put forward as a candidate for host-directed therapy of tuberculosis, but some caution is warranted. For example, in a model of candidemia, metformin resulted in increased lethality [36]. Also, it is unknown whether tuberculosis or concurrent use of antituberculous drugs increases the risk of metformin-associated gastrointestinal side effects or lactic acidosis [37]. With regard to possible drug interactions, a recent study in diabetic patients with tuberculosis has shown that rifampicin increases metformin exposure but does not alter blood glucose levels.

In summary, metformin effectively modulates the balance between inflammation and effective host responses to *M. tuberculosis*. It ameliorates the pathological inflammatory responses associated with tuberculosis while enhancing antimycobacterial processes such as ROS production and phagocytosis in humans.

## Supplementary Data

Supplementary materials are available at *The Journal of Infectious Diseases* online. Consisting of data provided by the authors to benefit the reader, the posted materials are not copyedited and are the sole responsibility of the authors, so questions or comments should be addressed to the corresponding author.

SummaryMetformin has shown beneficial effects in a murine model of tuberculosis. Using in-vitro and in-vivo studies we show that metformin has beneficial effects on cellular metabolism, immune function and gene-transcription involved in innate host responses to *M. tuberculosis* in humans.

jiz064_suppl_Supplementary_Figures

jiz064_suppl_Supplementary_Material

## Notes

***Acknowledgments*.** E. L. designed, performed, and analyzed the experiments; conducted the trial; and wrote the manuscript. C. E., J. M. C., and H. M. D. performed and analyzed the RNAseq data and wrote and reviewed the manuscript. V. K., B. B., R. J. W. A., and C. V. D. H. helped with the trial and performed experiments. J. B. and M. B. M. performed and analyzed mycobacterial killing and mitochondrial experiments. J. C., K. W. W. T., and E. N. performed and/or analyzed the CyTOF data. A.S. analyzed the mycobacterial experiments and CyTOF data and wrote the manuscript. M. G. N. and R. V. C. supervised the entire study, designed experiments, conducted the trial, analyzed data, and wrote the manuscript.

***Financial support*.** This study was supported by the European Union’s Seventh Framework Programme (to the TANDEM Consortium, under grant agreement 305279); the Singapore Immunology Center, Agency of Science, Technology, and Research (A*STAR; to A. S.); the A*STAR Joint Council Office (career development award 15302FG151 to A. S.); the European Research Council (consolidator grant 310372 to M. G. N.); and the Netherlands Organization for Scientific Research (Spinoza grant to M. G. N.).

***Potential conflicts of interest*.** A. S. holds the patent for the use of metformin to control mycobacterial infection (WO2014039011A1). All other authors report no potential conflicts. All authors have submitted the ICMJE Form for Disclosure of Potential Conflicts of Interest. Conflicts that the editors consider relevant to the content of the manuscript have been disclosed.
