# Supplementary material for: Metformin Alters Human Host Responses to Mycobacterium tuberculosis in Healthy Subjects
Source: J Infect Dis. 2019 Feb 12;220(1):139–50. doi: 10.1093/infdis/jiz064 (PMC6548897; doi:10.1093/infdis/jiz064)
Supplement: jiz064_suppl_Supplementary_Figures [file jiz064_suppl_supplementary_figures.pptx]

## Slide 1
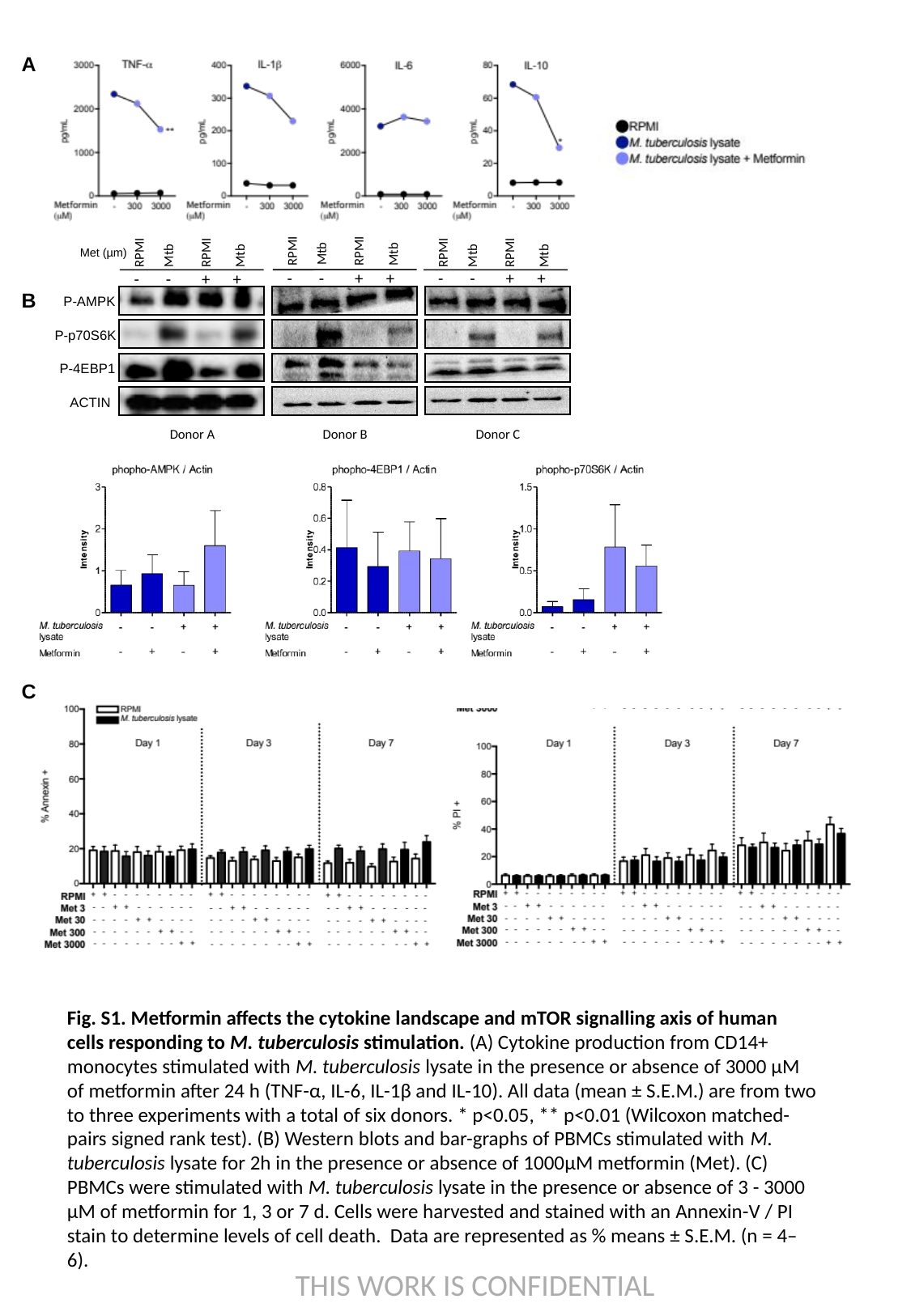

A
RPMI
RPMI
RPMI
RPMI
RPMI
RPMI
Mtb
Mtb
Mtb
Mtb
Mtb
Mtb
- - + +
- - + +
- - + +
P-AMPK
P-p70S6K
P-4EBP1
ACTIN
Donor A
Donor B
Donor C
Met (µm)
B
C
Fig. S1. Metformin affects the cytokine landscape and mTOR signalling axis of human cells responding to M. tuberculosis stimulation. (A) Cytokine production from CD14+ monocytes stimulated with M. tuberculosis lysate in the presence or absence of 3000 µM of metformin after 24 h (TNF-α, IL-6, IL-1β and IL-10). All data (mean ± S.E.M.) are from two to three experiments with a total of six donors. * p<0.05, ** p<0.01 (Wilcoxon matched-pairs signed rank test). (B) Western blots and bar-graphs of PBMCs stimulated with M. tuberculosis lysate for 2h in the presence or absence of 1000µM metformin (Met). (C) PBMCs were stimulated with M. tuberculosis lysate in the presence or absence of 3 - 3000 µM of metformin for 1, 3 or 7 d. Cells were harvested and stained with an Annexin-V / PI stain to determine levels of cell death. Data are represented as % means ± S.E.M. (n = 4–6).
THIS WORK IS CONFIDENTIAL

## Slide 2
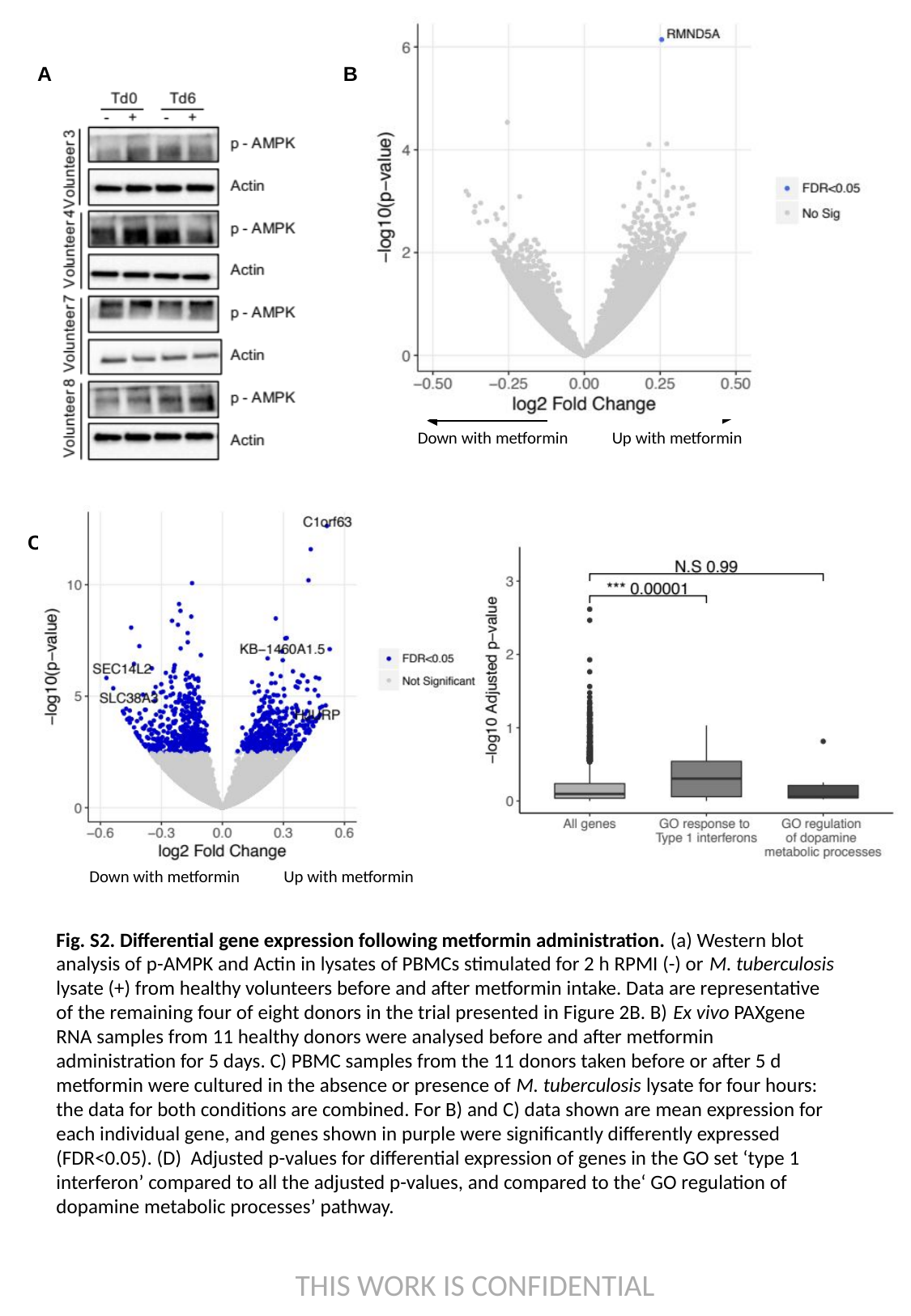

A
B
Down with metformin
Up with metformin
D
C
Down with metformin
Up with metformin
Fig. S2. Differential gene expression following metformin administration. (a) Western blot analysis of p-AMPK and Actin in lysates of PBMCs stimulated for 2 h RPMI (-) or M. tuberculosis lysate (+) from healthy volunteers before and after metformin intake. Data are representative of the remaining four of eight donors in the trial presented in Figure 2B. B) Ex vivo PAXgene RNA samples from 11 healthy donors were analysed before and after metformin administration for 5 days. C) PBMC samples from the 11 donors taken before or after 5 d metformin were cultured in the absence or presence of M. tuberculosis lysate for four hours: the data for both conditions are combined. For B) and C) data shown are mean expression for each individual gene, and genes shown in purple were significantly differently expressed (FDR<0.05). (D) Adjusted p-values for differential expression of genes in the GO set ‘type 1 interferon’ compared to all the adjusted p-values, and compared to the‘ GO regulation of dopamine metabolic processes’ pathway.
THIS WORK IS CONFIDENTIAL

## Slide 3
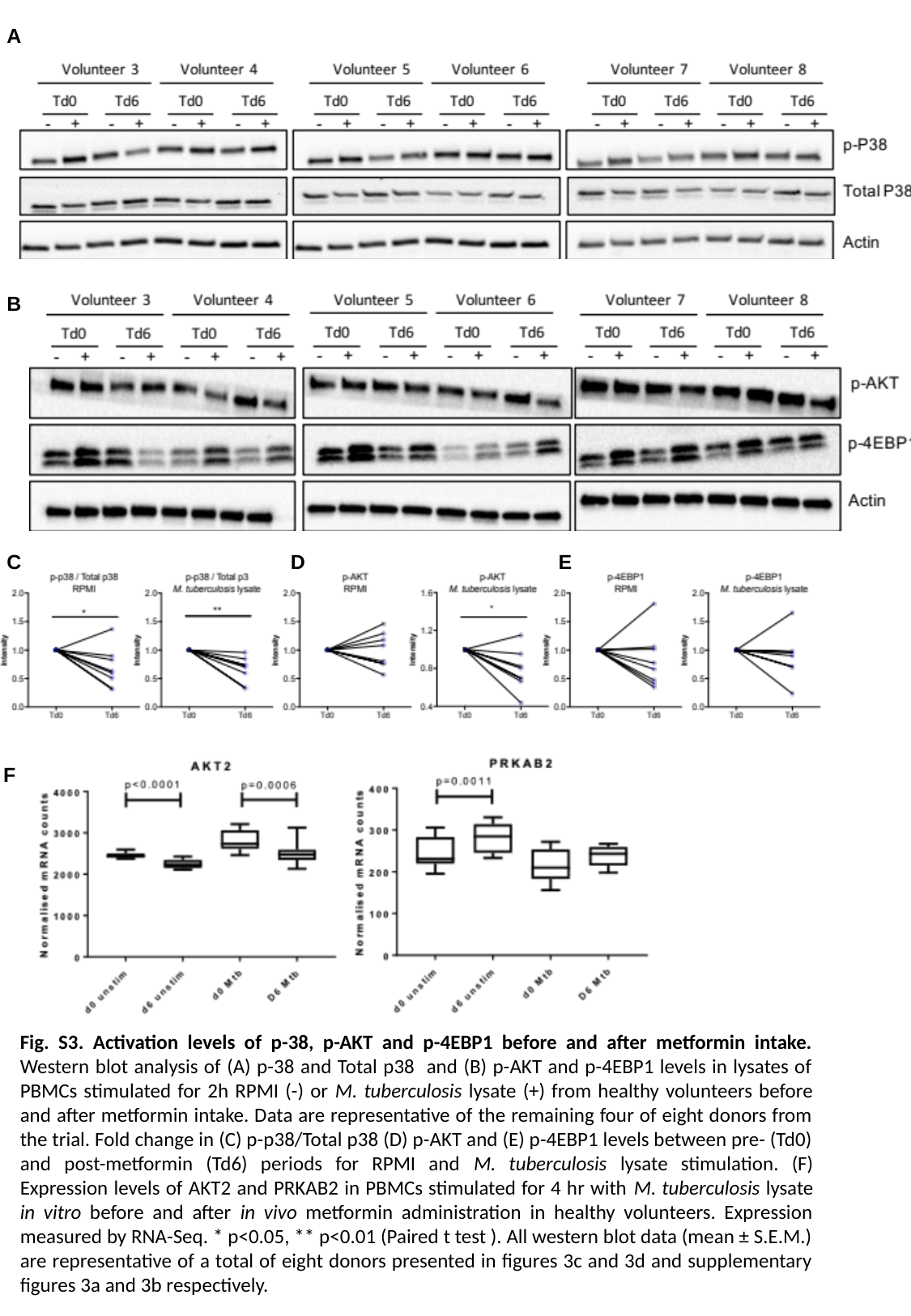

A
B
C
D
E
F
Fig. S3. Activation levels of p-38, p-AKT and p-4EBP1 before and after metformin intake. Western blot analysis of (A) p-38 and Total p38 and (B) p-AKT and p-4EBP1 levels in lysates of PBMCs stimulated for 2h RPMI (-) or M. tuberculosis lysate (+) from healthy volunteers before and after metformin intake. Data are representative of the remaining four of eight donors from the trial. Fold change in (C) p-p38/Total p38 (D) p-AKT and (E) p-4EBP1 levels between pre- (Td0) and post-metformin (Td6) periods for RPMI and M. tuberculosis lysate stimulation. (F) Expression levels of AKT2 and PRKAB2 in PBMCs stimulated for 4 hr with M. tuberculosis lysate in vitro before and after in vivo metformin administration in healthy volunteers. Expression measured by RNA-Seq. * p<0.05, ** p<0.01 (Paired t test ). All western blot data (mean ± S.E.M.) are representative of a total of eight donors presented in figures 3c and 3d and supplementary figures 3a and 3b respectively.

## Slide 4
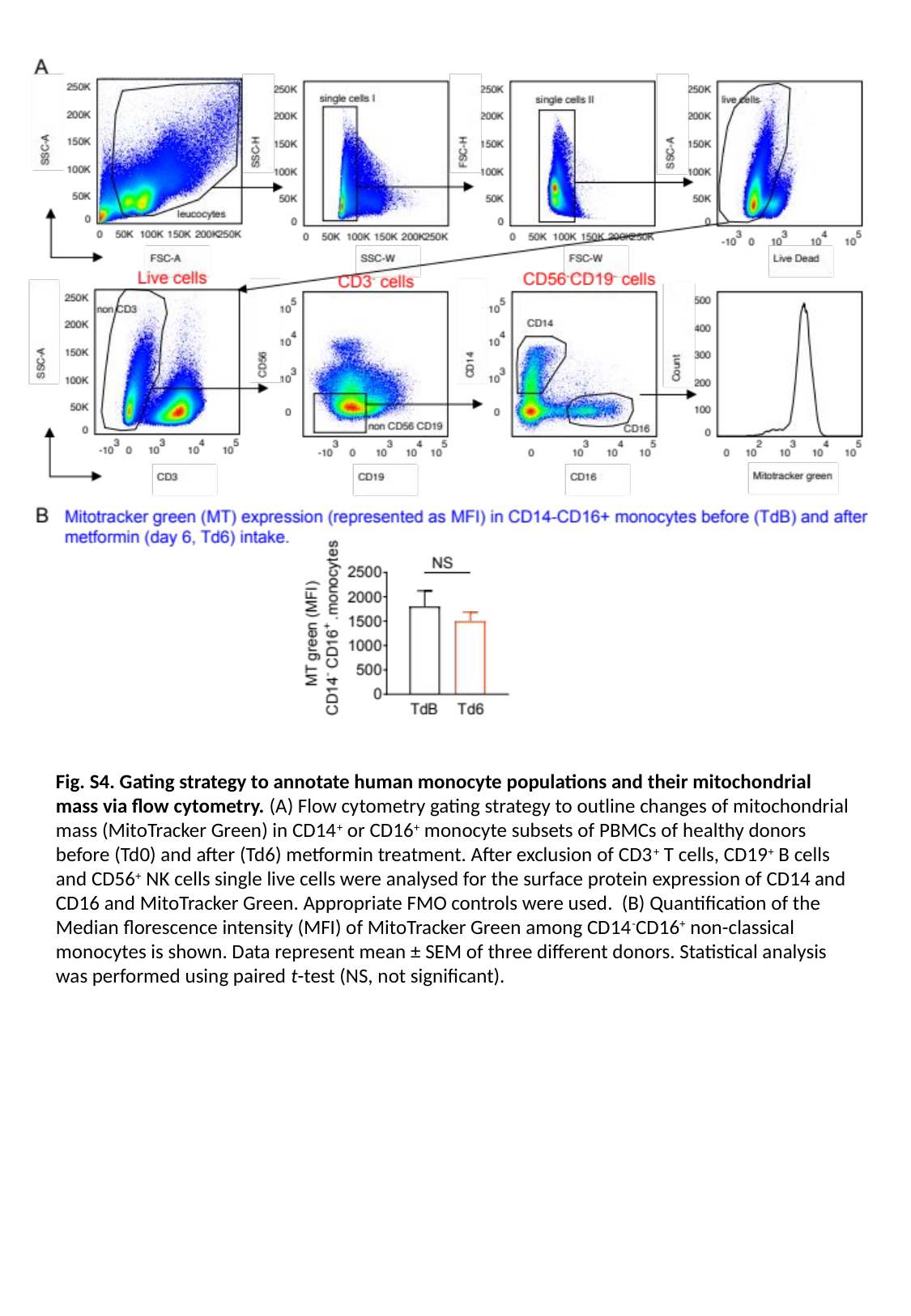

Fig. S4. Gating strategy to annotate human monocyte populations and their mitochondrial mass via flow cytometry. (A) Flow cytometry gating strategy to outline changes of mitochondrial mass (MitoTracker Green) in CD14+ or CD16+ monocyte subsets of PBMCs of healthy donors before (Td0) and after (Td6) metformin treatment. After exclusion of CD3+ T cells, CD19+ B cells and CD56+ NK cells single live cells were analysed for the surface protein expression of CD14 and CD16 and MitoTracker Green. Appropriate FMO controls were used. (B) Quantification of the Median florescence intensity (MFI) of MitoTracker Green among CD14-CD16+ non-classical monocytes is shown. Data represent mean ± SEM of three different donors. Statistical analysis was performed using paired t-test (NS, not significant).

## Slide 5
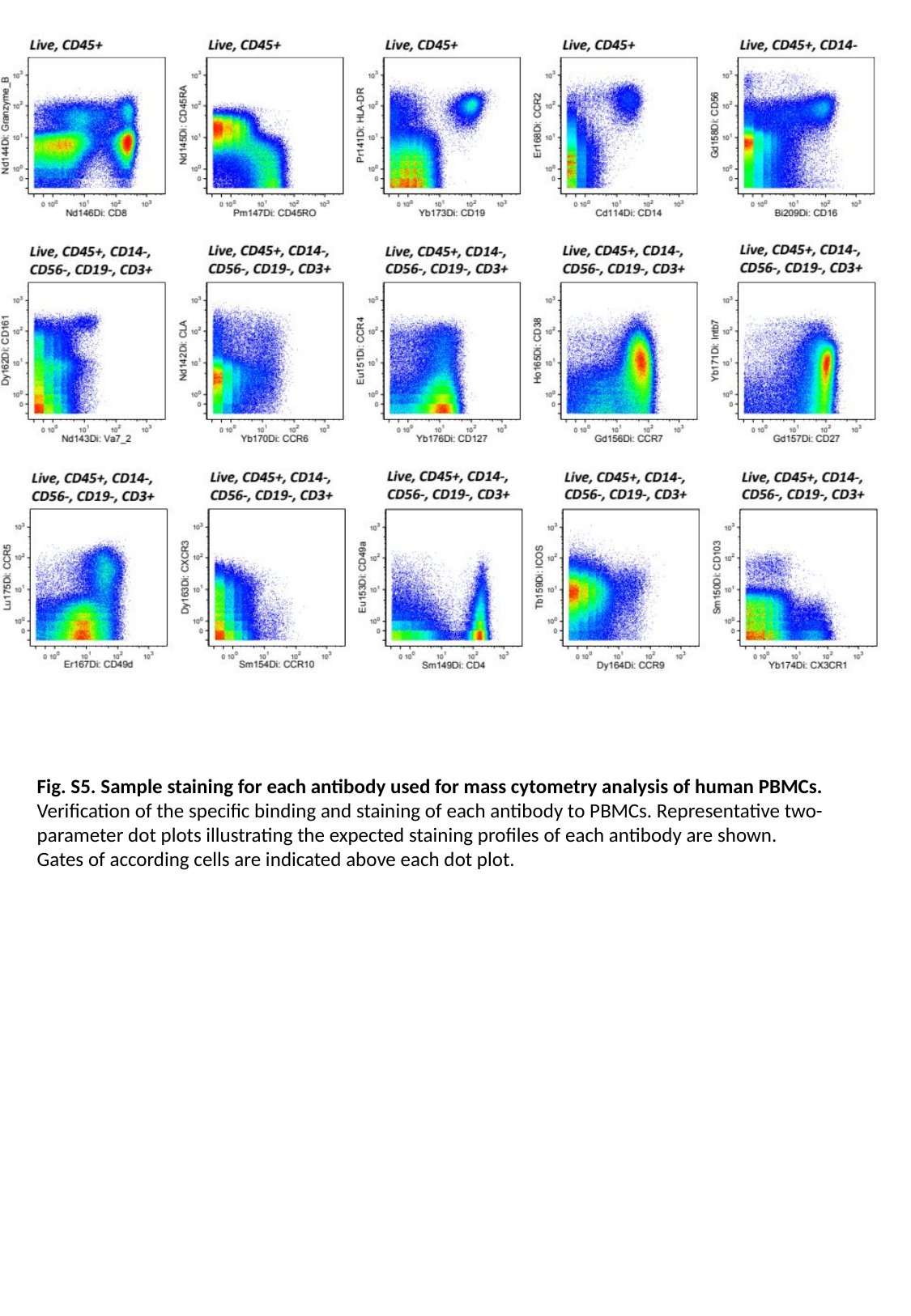

Fig. S5. Sample staining for each antibody used for mass cytometry analysis of human PBMCs. Verification of the specific binding and staining of each antibody to PBMCs. Representative two-parameter dot plots illustrating the expected staining profiles of each antibody are shown. Gates of according cells are indicated above each dot plot.

## Slide 6
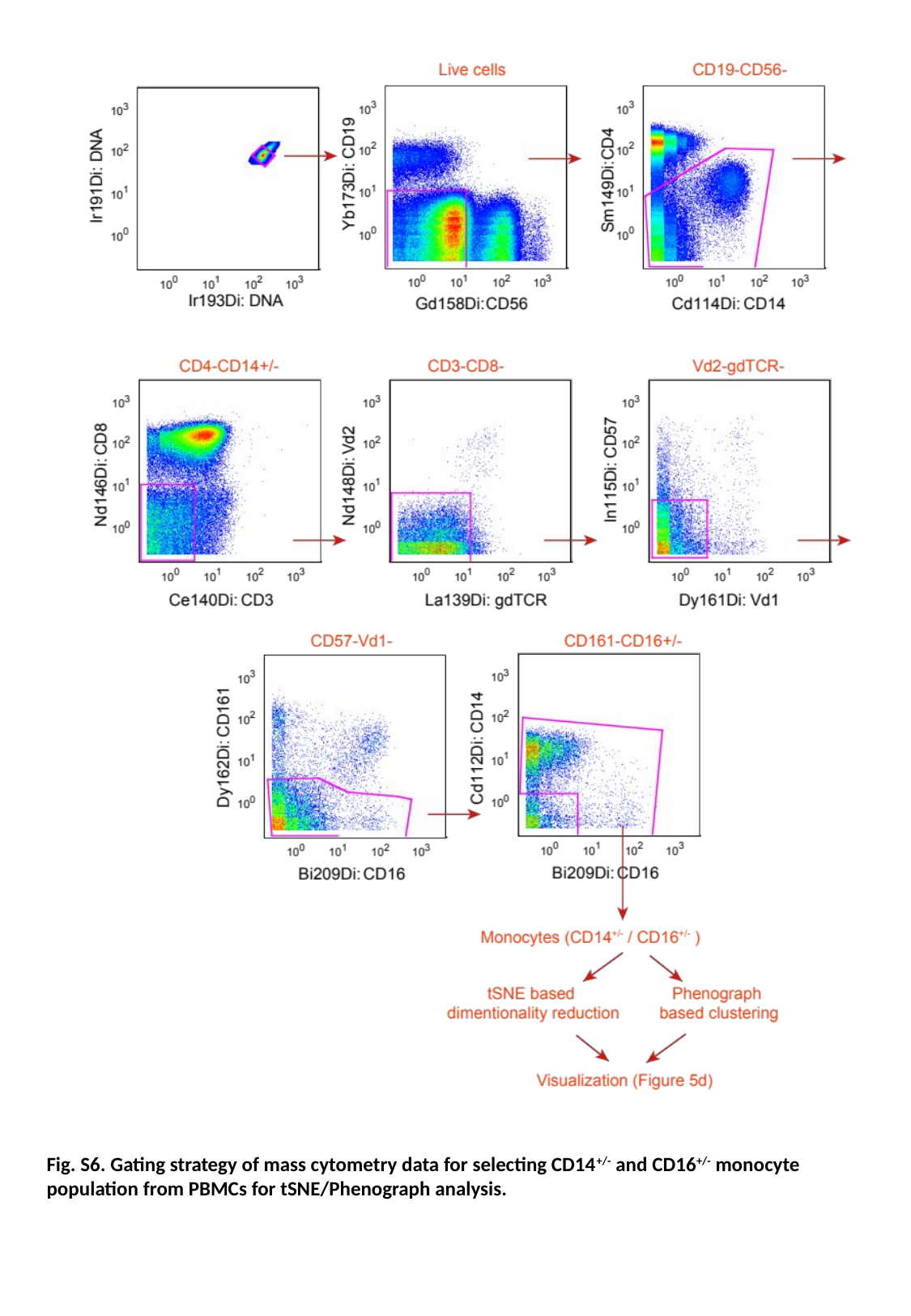

Fig. S6. Gating strategy of mass cytometry data for selecting CD14+/- and CD16+/- monocyte population from PBMCs for tSNE/Phenograph analysis.

## Slide 7
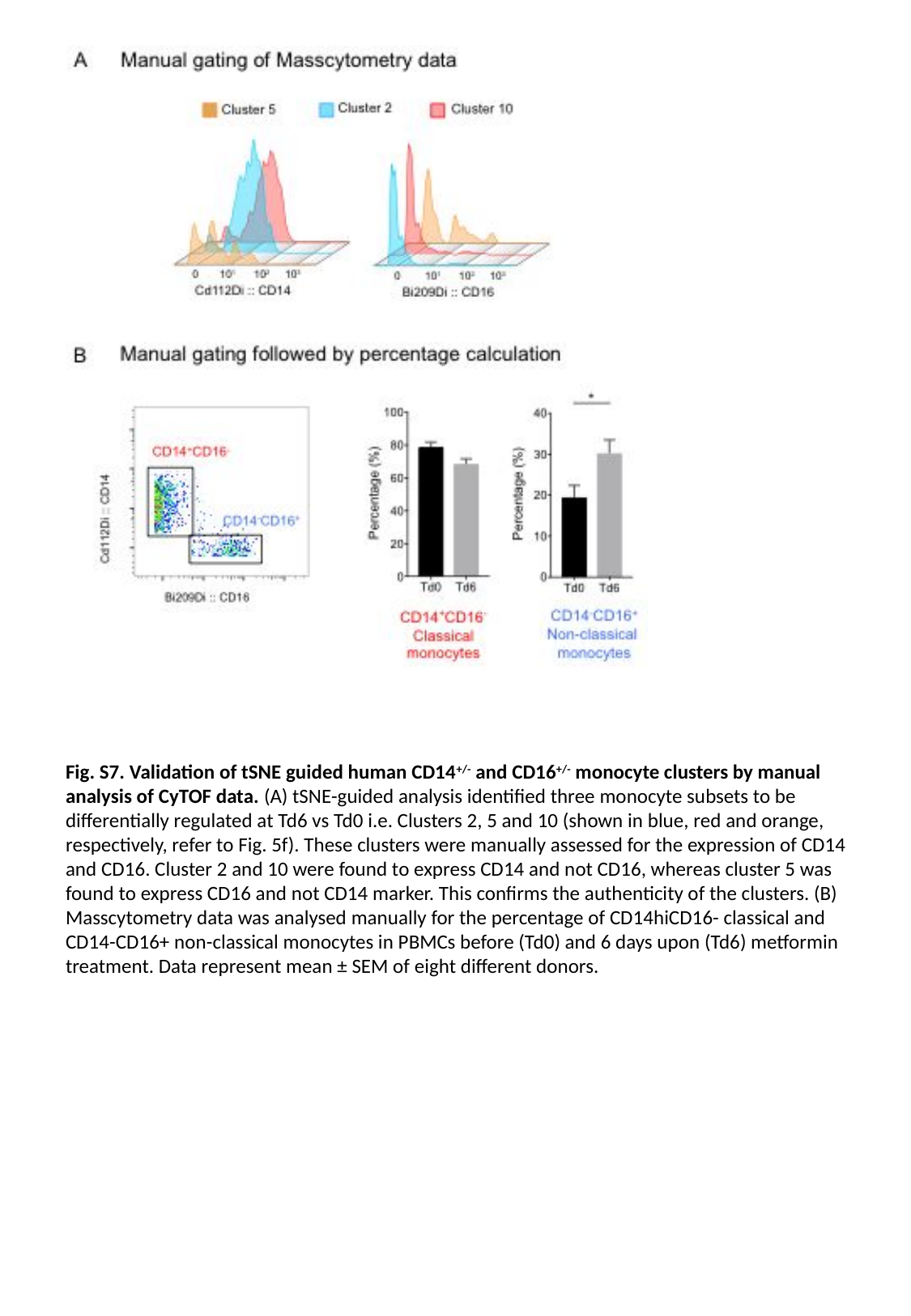

Fig. S7. Validation of tSNE guided human CD14+/- and CD16+/- monocyte clusters by manual analysis of CyTOF data. (A) tSNE-guided analysis identified three monocyte subsets to be differentially regulated at Td6 vs Td0 i.e. Clusters 2, 5 and 10 (shown in blue, red and orange, respectively, refer to Fig. 5f). These clusters were manually assessed for the expression of CD14 and CD16. Cluster 2 and 10 were found to express CD14 and not CD16, whereas cluster 5 was found to express CD16 and not CD14 marker. This confirms the authenticity of the clusters. (B) Masscytometry data was analysed manually for the percentage of CD14hiCD16- classical and CD14-CD16+ non-classical monocytes in PBMCs before (Td0) and 6 days upon (Td6) metformin treatment. Data represent mean ± SEM of eight different donors.

## Slide 8
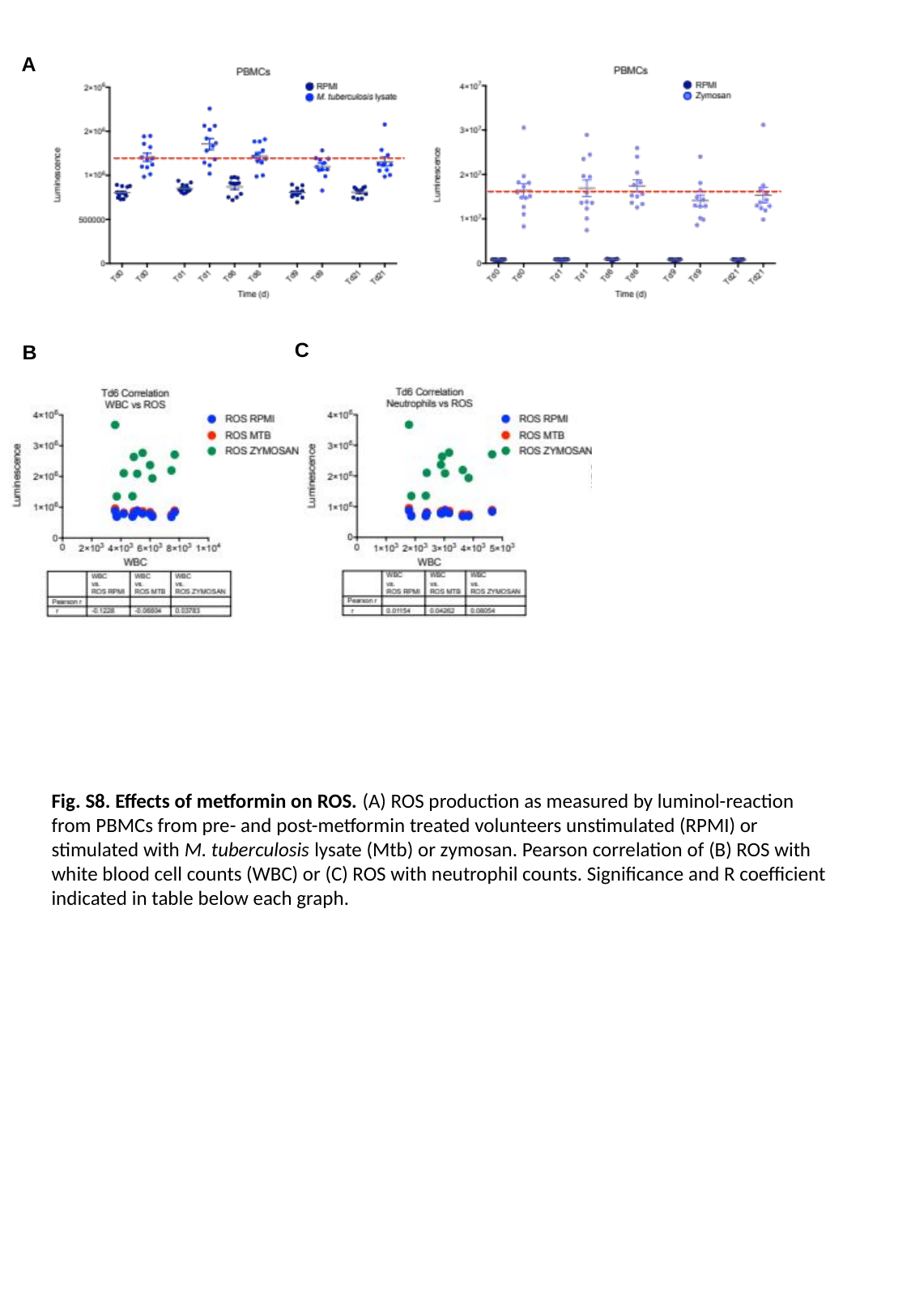

A
C
B
Fig. S8. Effects of metformin on ROS. (A) ROS production as measured by luminol-reaction from PBMCs from pre- and post-metformin treated volunteers unstimulated (RPMI) or stimulated with M. tuberculosis lysate (Mtb) or zymosan. Pearson correlation of (B) ROS with white blood cell counts (WBC) or (C) ROS with neutrophil counts. Significance and R coefficient indicated in table below each graph.

## Slide 9
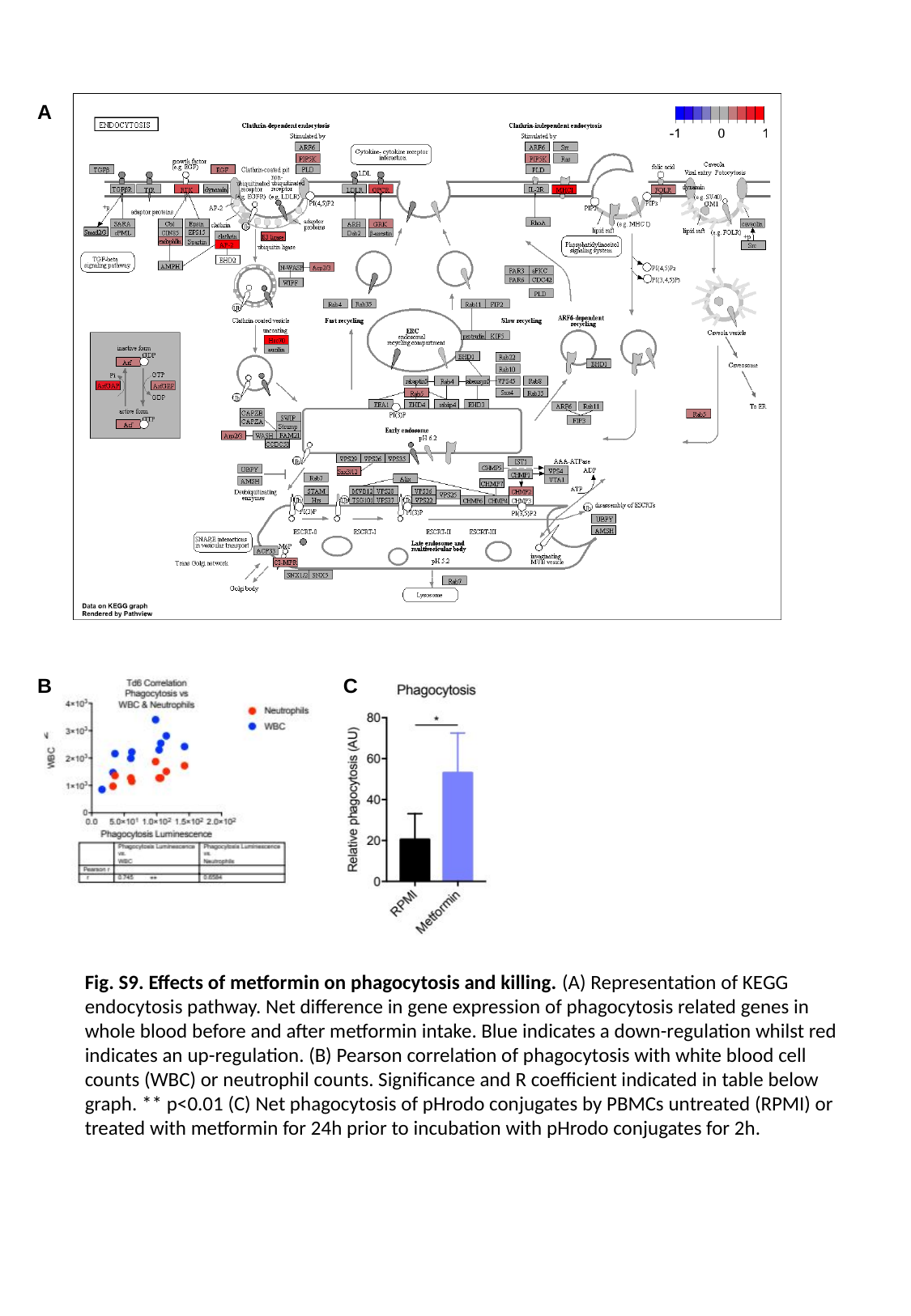

A
B
C
C
Fig. S9. Effects of metformin on phagocytosis and killing. (A) Representation of KEGG endocytosis pathway. Net difference in gene expression of phagocytosis related genes in whole blood before and after metformin intake. Blue indicates a down-regulation whilst red indicates an up-regulation. (B) Pearson correlation of phagocytosis with white blood cell counts (WBC) or neutrophil counts. Significance and R coefficient indicated in table below graph. ** p<0.01 (C) Net phagocytosis of pHrodo conjugates by PBMCs untreated (RPMI) or treated with metformin for 24h prior to incubation with pHrodo conjugates for 2h.
