# Supplementary material for: Metformin Alters Human Host Responses to Mycobacterium tuberculosis in Healthy Subjects
Source: J Infect Dis. 2019 Feb 12;220(1):139–50. doi: 10.1093/infdis/jiz064 (PMC6548897; doi:10.1093/infdis/jiz064)
Supplement: jiz064_suppl_Supplementary_Material [file jiz064_suppl_supplementary_material.docx]

**ONLINE DATA SUPPLEMENT**

**Supplementary Materials & Methods Titles**

- Study subjects
- Cellular isolation and differentiation
- Western Blot Measurements.
- Cytokine production.
- Cellular proliferation
- Metabolic measurements.
- Reactive Oxygen Species Measurements.
- Phagocytosis.
- *M. tuberculosis* cellular infection
- Cellular Viability
- RNA-Seq and qRT-PCR transcriptomics analyses
- CyTOF marker labeling and data acquisition.
- Mass cytometry data analysis.

**Supplementary Table Titles**

- Table S1. Antibodies used for mass cytometry analysis of lung myeloid cells. Metal conjugate, antibody clone name and supplier of each marker has been listed.
- Table S2. Primer Sequences

**Supplementary Figure Titles**

Fig. S1. Metformin affects the cytokine landscape of human cells responding to *M. tuberculosis* stimulation.

Fig. S2. Differential gene expression following metformin administration

Fig. S3. Activation levels of p-38, p-AKT and p-4EBP1 before and after metformin intake

Fig. S4. Gating strategy to annotate human monocyte populations and their mitochondrial mass via flow cytometry.

Fig. S5. Sample staining for each antibody used for mass cytometry analysis of human PBMCs.

Fig. S6. Gating strategy of mass cytometry data for selecting CD14+/- and CD16+/- monocyte population from PBMCs for tSNE/Phenograph analysis.

Fig. S7. Validation of tSNE guided human CD14+/- and CD16+/- monocyte clusters by manual analysis of CyTOF data.

Fig. S8. Effects of metformin on ROS.

Fig. S9. Effects of metformin on phagocytosis and killing.

**Supplementary Materials & Methods**

*Study subjects. In vitro* experiments were performed with peripheral blood mononuclear cells (PBMCs) isolated from buffy coats obtained from healthy volunteers (Sanquin Blood bank, Nijmegen, The Netherlands). As donations were anonymous, no tuberculosis skin tests or IFN-γ release assay was performed. The incidence of tuberculosis in the Dutch population is extremely low (1.5/100,000), and Bacillus Calmette-Guérin (BCG) vaccination is not part of the routine vaccination program. To study the *in vivo* effect of metformin, 11 male healthy non-obese volunteers without medication, kidney function loss or metabolic disorders, were asked to take metformin in increasing doses starting at 500 mg once a day and ending with 1000 mg twice a day. Blood was drawn at two baseline time points: 1 d before and immediately before metformin intake, and again immediately after, 3 d after and 2 weeks after the last dose of metformin. Experiments were conducted according to the principles expressed in the Declaration of Helsinki. Both for the *in vitro* (NL32357.091.10) and healthy volunteers (NL47793.091.14) studies ethical approval was granted by the Arnhem-Nijmegen Ethical Committee. As validation EDTA blood from 10 healthy young subjects given metformin (500 mg day 1-2) increasing to 1000 mg (day 3-8) was examined as part of a pharmacokinetic study (NL53534.091.15).

*Cellular isolation and differentiation.* Isolation of PBMCs was performed by differential centrifugation over Ficoll-Paque™ PLUS (GE Healthcare Biosciences). CD14^+^ monocytes were purified from isolated PBMCs using MACS microbeads for positive selection, according to the manufacturer’s instructions (Miltenyi Biotec). To generate macrophages, 3 x 10^7^ PBMCs were incubated at 37°C in petri dishes (Corning) for 1 h. Non–adherent cells were then washed away using warm PBS three times. The remaining adherent monocytes were differentiated into M1 or M2 macrophages in 10% human pooled serum and 5 ng/mL GM-CSF (R&D Systems) or 50 ng/mL M-CSF (R&D Systems) respectively for 6 d. Media containing growth factors and serum were refreshed on day 3 of differentiation. At 6 d post differentiation, adherent macrophages were harvested using Versene Solution (Thermo Fisher Scientific). All cell types were re-suspended in RPMI^+^ (RPMI 1640 (Gibco) supplemented with 10 𝜇g/mL gentamicin (Lonza), 10 mM L-glutamine (Life Technologies), and 10mM pyruvate (Life Technologies). Cells were counted in a Coulter counter (Coulter Electronics) and adjusted to 5 x 10^6^ PBMCs/mL, 1 x 10^6^ CD14^+^ monocytes/mL or 1 × 10^6^ macrophages/mL.

*Cytokine production.* 100 μL of PBMCs, CD14^+^ monocytes or 75 μL of M1 / M2 macrophages were stimulated in RPMI^+^ with or without 1 - 5 μg/mL *Mycobacterium tuberculosis* strain H37Rv (*M. tuberculosis*) lysate, in the presence or absence of 3 – 3000 μM Metformin (Sigma-Aldrich) for 4 h, 24 h or with 10% pooled human serum for 7 days. Cell culture supernatants were collected and stored at −20°C. Cytokines in culture supernatants were measured by commercial ELISA kits: interleukin (IL)-1β, tumour necrosis factor (TNF)-α, IL-17A, IL-22 (R&D Systems) and IL-6, interferon gamma (IFN-γ) and IL-10 (Sanquin), after diluting supernatants as necessary.

*Cellular proliferation.* Proliferation of PBMCs was measured using the CFSE kit (BioLegend) according to the instructions described by the manufacturer. Briefly, PBMCs were re-suspended at a density of 10 x 10^6^ cells/mL in PBS and labelled by adding CFSE in a 1: 1 ratio at a final concentration of 1.25 µM. The suspension was mixed gently and incubated for 5 min at 37°C. An equal volume of 100% human pooled serum was added and incubated for 3 min at room temperature. Cells were washed twice in RPMI supplemented with 10% human pooled serum and re-suspended to a concentration of 5 x 10^6^ PBMCs/mL in RPMI^+^. An unlabelled cell fraction and a labelled cell fraction were measured by flow cytometry on day 0 to determine staining efficacy. Labelled cells were stimulated for 6 d with *M. tuberculosis* lysate in the presence or absence of 300 µM metformin. On day 6, cells were stained with anti-CD4 (PE-Cy5 conjugated, ITK Diagnostics BV) and measured by flow cytometry. The percentage of proliferated CD4^+^ cells was calculated as percentage of all CD4^+^ cells.

*Metabolic measurements*. Lactate was measured from stored cell culture supernatants using a coupled enzymatic assay in which lactate was oxidised and the resulting H_2_O_2_ was coupled to the conversion of Amplex® Red reagent to fluorescent resorufin by HRP (horseradish peroxidase) (1). Measurement of the NAD^+^/NADH redox ratio was adapted from Zhu et al (2). Glucose consumption was measured according to the manufacturer’s instructions using the Amplex® Red Glucose/Glucose Oxidase Assay Kit (Life Technologies). Measurement of mitochondrial mass and potential (using MitoTracker Green and Deep Red, Life Technologies) by flow cytometry.

*Western Blot Measurements*. Pellets of 5 x 10^6^ PBMCs per condition were lysed in 100 μL lysis buffer (1M Tris pH 7.4, 0.5M EDTA, 5M NaCl, 10% ND40, 0.5M NaF, 2.5% sodium deoxycholate, PhosSTOP (Roche) and cOmplete (Roche)). The cell homogenate was frozen, thawed and processed for Western blot analysis according to the manufacturer’s instructions. Western blotting was carried out using Mini-PROTEAN TGX precast Gels (Bio-Rad). Proteins were transferred using the Trans-Blot^®^ Turbo™ system (Bio-Rad) according to the manufacturer’s instructions. Blots were incubated overnight at 4°C with actin at 1:1000, phospho-AMPK (T172) (p-AMPK) at 1:500, phospho p70 S6K (T389) (p-p70 S6K) at 1:500, phospho-4EBP1 (T37/460) (p-4EBP1) at 1:1000, phospho-P38 (T180/Y182) (p-P38) at 1:1000, total-P38 at 1:000 or phospho-AKT (S473) (p-AKT) at 1:1000. Actin was bought from Sigma and all other antibodies from Cell Signalling. Secondary antibody used was swine anti-rabbit at 1:5000 (Dako). SuperSignal West Femto Substrate (Thermo Fisher Scientific) or ECL (Bio-Rad) were used for visualisation of proteins.

*Reactive Oxygen Species Measurements*. 100 𝜇L of whole blood diluted 100x in Hanks' buffered salt solution (HBSS) or 50 𝜇L of a total of 2.5 x 10^5^ PBMCs together with 50 𝜇L of HBSS was added in quadruplicate to each well of a white 96-well assay plate (Corning). Cells were incubated with 50 𝜇L of 1 mg/mL serum-opsonized zymosan, 50 𝜇L of 10 𝜇g/mL serum-opsonised *M. tuberculosis* lysate or 50 𝜇L of 100% human pooled serum as control. 50 𝜇L of 145 𝜇g/mL luminol (Sigma) was added, and chemiluminescence was measured every 142 s for 1 h. Opsonized zymosan particles were prepared by incubation of zymosan derived from *Saccharomyces cerevisiae* (Sigma-Aldrich) in pooled human serum for 30 min at 37°C, after which the particles were washed twice in PBS and re-suspended in PBS. Opsonised *M. tuberculosis* lysate was prepared by incubation of lysate in 100% human pooled serum for 1 h prior to making aliquots and freezing.

*Phagocytosis.* pHrodo® Green Zymosan Bioparticles® Conjugate (Thermo Fisher Scientific) was used to measure the rate of phagocytosis. The pH-sensitive molecular probes are almost non-fluorescent in a neutral environment, but the dye lights up when the pH decreases in the lysosome. So the amount of emitted fluorescence is a relative measure for the rate of phagocytosis. One vial of pHrodo particles was dissolved in 2 mL RPMI and sonicated for 5 min. *For in vitro measurements*, 5 x 10^5^ PBMCs were incubated for 24 h at 37°C in a flat bottom black plate with RPMI or 1000 𝜇M metformin. The supernatant was removed and 100 𝜇l RPMI (cells only control) or pHrodo suspension was added to each well in duplicate and incubated for 2 h in a non-CO_2_ elevated incubator at 37°C before measuring fluorescence. For the trial volunteers, peripheral blood leukocytes were isolated by lysis of erythrocytes using hypotonic lysis buffer (155 mM NH_4_Cl, 10 mM KHCO_3_). After isolation, the cells were rested for 30 min at 37°C in a black 96-wells plate, after which RPMI (cells only control) or pH rodo suspension was added to each well in duplicate and incubated for 2 h in a non-CO_2_ elevated incubator at 37°C before measuring fluorescence. Fluorescence was measured at an excitation of 486 nm and an emission rate of 528 nm.

*M. tuberculosis cellular infection.* Frozen mycobacteria, H37Rv, were thawed, washed and re-suspended in antibiotic-free RPMI 1640 with 10% fetal bovine serum (FBS) and were used to infect 3 x 10^6^ PBMCs in 15 mL falcon tubes with a multiplicity of infection (MOI) of 5. The infected cells were incubated at 37°C with 5% CO_2_ for 3 h. After this time, cells were washed two times with antibiotic-free medium by centrifuging at 800 rpm. The infected cells were counted and seeded in triplicate for 3, 24, or 48 h. At predetermined time points after infection, the infected cells were washed once with PBS and then lysed with 200 mL of PBS with 1% SDS. Various dilutions of this lysate were plated on Middlebrook 7H11 agar supplemented with 10% oleic acid–albumin– dextrose–catalase (OADC, Difco Laboratories), in triplicate. Agar plates were incubated at 37°C for 3 weeks, after which colonies were counted visually. CFUs obtained from two or three dilutions were used to calculate the total number of CFU per mL.

*Cellular Viability.* To examine early or late apoptosis, 5 x 10^5^ treated PBMCs were washed with PBS, re-suspended in 200 μL of RPMI and incubated on ice in the dark with 1 μL of Annexin V-FITC (FITC, Biovision) for 15 min followed by a 5 min incubation with 1.5 μL of propidium-iodide (PI, Sigma Aldrich). The relative level of apoptotic cells was detected by flow cytometry within 1 h, using a FC500 flow cytometer (Beckman Coulter) and data were analysed using Kaluza 1.3 software (Beckman Coulter).

*RNA-Seq and qRT-PCR Transcriptomics:* Whole blood was captured in PAXgene RNA tubes (PreAnalytix) and RNA isolated using the PAXgene blood miRNA kit (Qiagen). RNA from isolated PBMCs and purified CD14^+^ cells was extracted using TRIzol reagent (Invitrogen), while cultured PBMC samples were frozen in RNAprotect (Qiagen) and RNA then extracted using the RNeasy Mini Kit (Qiagen). RNA was reverse-transcribed into complementary DNA by using either the iScript cDNA synthesis kit (BIORAD) or the Superscript IV VILO (Invitrogen). Quantitative real-time PCR (qPCR) primer sequences are listed in supplementary Table S2. Power SYBR Green PCR Master Mix (Applied Biosystems, Life technologies) was used for qPCR in an AB Step one plus or 7500 Fast real-time PCR system (Applied Biosystems) or the CFX384 Real-Time PCR Detection System (Biorad). qPCR data were normalized to the housekeeping gene human β2M or HuP0.

For RNA-sequencing, RNA was quantified using the LabChip GX HiSens RNA system (Perkin Elmer). RNA from the whole blood Paxgene samples was depleted of globin transcripts using GLOBINclear ^TM^ (Invitrogen). Whole blood samples were processed using the Ribo-Zero TruSeq stranded total RNA library preparation method (Illumina) whereas cultured PBMC RNA samples were processed using the TruSeq stranded mRNA library prep kit (Illumina). All samples were sequenced on a NextSeq500, generating ~36-45M million 43bp paired-end reads per sample. The data are deposited at NCBI GEO accession number: GSE102678. FASTQ sequence files were aligned to the human genome version Human_g1k_v37 using STARAligner (3) and aligned reads which overlapped with 63,677 genome features were counted using HTSeq-count with annotation version Homo_sapiens.GRCh37.75.gtf (4). Differentially expressed genes were calculated using the R package DESeq2 (5) in Bioconductor, with false discovery rate correction applied for multiple testing. Gene set analyses were performed on the entire datasets using the Stouffer method in the Piano R package (6) with the MSigDB Hallmark gene sets database (7) as well as the KEGG pathways database. The human RNA Seq study was approved by the LSHTM Research Ethics Committee (#11968).

*CyTOF marker labeling and data acquisition***.** PBMCs cells were thawed and cultured for 2 h before stimulating them with PMA and inomycin (1 x cocktail) for 4 h in a 96-well U-bottom plate (BD Falcon). Cells were then first washed twice in cold 1 x PBS (Gibco) and incubated on ice with 200 μM cisplatin (Sigma-Aldrich) for 5min. Cells were then washed and stained with anti-Mertk-biotin (1:50) for 30 min on ice, washed twice with FACS buffer (1 x PBS + 4% FBS + 0.05% Azide) and stained with heavy-metal isotope-labeled antibodies on ice. Purified antibodies used for mass cytometry analysis were purchased from companies listed in Table S1. Antibody conjugation was performed as previously described (8, 9). After 30 min, cells were washed twice in FACS buffer and once in PBS, and then fixed in PBS containing 2% paraformaldehyde (Electron Microscopy Sciences) at 4°C. The next day, cells were washed twice in permeabilization (perm) buffer (BioLegend). After washing in 1 x PBS, each sample was stained with a unique, dual combination of barcodes for 30 min on ice. Cells were then washed once with perm buffer, followed by incubation in FACS buffer for 10 min on ice. Cellular DNA was labeled at room temperature using 250 nM Iridium Interchelator (Fluidigm), diluted in 2% PFS/PBS (1:2000), for 20 min. Cells were then washed twice with FACS buffer and twice with distilled water before final resuspension in distilled water. Bromoacetamidobenzyl-EDTA (BABE)- and DOTA-maleimide (DM)-linked metal barcodes were prepared as previously described (8). Two barcoded batches were prepared, each having an equal total number of samples. For each batch, a small aliquot of cells were taken from randomly selected wells and combined as an internal control. In each batch, cells from all samples were pooled, counted, and diluted to a final concentration of 0.5 × 10^6^ cells/ml for acquisition on CyTOF 1 (Fluidigm). After acquisition, the data were exported in a flow-cytometry-file (.fcs) format. Each sample with a unique combination of two barcodes was de-barcoded using manual gating in FlowJo.

*Mass cytometry data analysis***.** A single composite map representing the phenotypic diversity of all monocyte cells derived from all analyzed samples was created, which allowed a comparison of the cellular composition between individuals / treatment group to be performed. FCS files of live CD14^+^CD16^+^ monocytes were converted to intensity values using R package flowCore. The intensity values were logicle transformed (using parameters w = 0.1, *t* = 4000, m = 4.5, a = 0), and markers (fig. E6) were selected for downstream analysis. After separately gating and exporting monocyte cells from each sample, the aggregate population of cells was subjected to tSNE dimension reduction. tSNE was carried out using bh_tsne, an efficient implementation of t-SNE for large data sets (obtained from Laurens van der Maaten). The original marker expression values before t-SNE dimension reduction were then fed to Phenograph clustering algorithm (10). Phenograph clustering was performed using cytofkit R package (11).

A scatter plot of cells on the 2D tSNE dimensions was generated, with each cell color-coded by Phenograph clusters using SpotFire. Cells were grouped by Phenograph clusters and median intensity values of markers were calculated for each cluster and visualized via plotting heat maps. The heat maps were used to identify defining markers for these clusters and annotate each of them as one of previously defined or unknown cellular populations. For each sample, the number of cells in different Phenograph clusters were counted and the percentage frequency of each cluster was calculated. Heat map and hierarchical clustering dendrogram were produced to compare the frequencies of Phenograph clusters in different samples. Mann Whitney test was performed on cluster frequencies to identify clusters that showed significantly different frequencies in samples before and after metformin intake (Td0 versus Td6). Phenograph cluster delineation was coded into a two-dimensional coordinate system that was then inverse-logicle transformed. Similarly, the coordinates of the 2D t-SNE map were also inverse-logicle transformed. The transformed cluster coordinates and t-SNE coordinates were added to the FCS files as additional parameters. Subsequently, we gated on the new FCS files and overlaid the gated subsets on the 2D t-SNE plot using FlowJo software (Tree Star Inc.).

**Supplementary Tables**

**Table S1.** Table of antibodies used for mass cytometry analysis of human PBMCs. Metal conjugate, antibody clone name and supplier of each marker has been listed.

| **Isotope Tag** | **Antibody** | **Clone** | **Company** |
| --- | --- | --- | --- |
| Y-89 | CD45 | HI30 | Fluidigm |
| Qdot (Cd112/114) | CD14 | TüK4 | Invitrogen |
| In-115 | CD57 | HCD57 | Biolegend |
| La-139 | Gamma delta (γδ) TCR | PE anti-human γδ TCR: 5A6.E9  Anti-PE: PE001 | PE anti-human γδ TCR: Invitrogen  Purified anti-PE: Biolegend |
| Ce-140 | CD3 | UCHT1 | Biolegend |
| Pr-141 | HLA-DR | L243 | Biolegend |
| Nd-142 | TNF-α | Mab11 | eBioscience |
| Nd-143 | IFN-γ | 4S.B3 | eBioscience |
| Nd-144 | MIP-1β | D21-1351 | BD Biosciences |
| Nd-145 | IL-8 | E8N1 | Biolegend |
| Nd-146 | CD8α | SK1 | Biolegend |
| Sm-147 | CD45RA | HI100 | Biolegend |
| Nd-148 | CD19 | HIB19 | Biolegend |
| Sm-149 | CD4 | SK3 | Biolegend |
| Nd-150 | CD103 | B-Ly7 | eBioscience |
| Eu-151 | IL-2 | MQ1-17H12 | eBioscience |
| Sm-152 | CD25 | M-A251 | Biolegend |
| Eu-153 | CD107a | H4A3 | BD Biosciences |
| Sm-154 | CCR7 | 150503 | R&D Systems |
| Gd-155 | ICOS | C398.4A | Biolegend |
| Gd-156 | Vδ2 | B6 | Biolegend |
| Gd-157 | CD38 | HIT2 | Biolegend |
| Gd-158 | CD56 | NCAM16.2 | BD Biosciences |
| Tb-159 | Integrin β7 | FIB504 | Biolegend |
| Gd-160 | PD-1 | eBioJ105 | eBioscience |
| Dy-161 | CCR9 | L053E8 | Biolegend |
| Dy-162 | CTLA-4 | BN13 | BD Biosciences |
| Dy-163 | CD40L | 24-31 | eBioscience |
| Dy-164 | Vδ1 | FITC anti-human Vδ1: REA173  Anti-FITC: FIT22 | FITC anti-human Vδ1: Miltenyi Biotec  Purified anti-FITC: Biolegend |
| Ho-165 | Vα7.2 | 3C10 | Biolegend |
| Er-166 | CXCR5 | RF8B2 | BD Biosciences |
| Er-167 | CD161 | HP-3G10 | Biolegend |
| Er-168 | CCR2 | K036C2 | Biolegend |
| Tm-169 | IL-4 | MP4-25D2 | Biolegend |
| Er-170 | IL-10 | JES3-9D7 | eBioscience |
| Yb-171 | CCR6 | G034E3 | Biolegend |
| Yb-172 | GM-CSF | BVD2-21C11 | Biolegend |
| Yb-173 | CCR4 | 205410 | R&D Systems |
| Yb-174 | IL-22 | Poly5161 | Biolegend |
| Lu-175 | CCR5 | HEK/1/85a | Abcam |
| Yb-176 | IL-17A | BL168 | Biolegend |
| Bi-209 | CD16 | 3G8 | Fluidigm |

Pre-stain antibodies

**Table S2.** Table of primer sequences used antibodies used in this study.

| Gene | Figure | Forward | Reverse |
| --- | --- | --- | --- |
| IL-18 | 1C | 5' - TGTCGCAGGAATAAAGATGGCT - '3 | 5' - CCTTGGTCAATGAAGAGAACTTGGT - '3 |
| IL-23 subunit p19 | 1C | 5' - CTCAGGGACAACAGTCAGTTC - '3 | 5' - ACAGGGCTATCAGGGAGC - '3 |
| IL12 subunit p35 | 1C | 5' - CCTTGCACTTCTGAAGAGATTGA - '3 | 5' - ACAGGGCCATCATAAAAGAGGT - '3 |
| TGF- β1 | 1C | 5' - CAATTCCTGGCGATACCTCAG - '3 | 5' - GCACAACTCCGGTGACATCAA - '3 |
| IFIT1 | 4A | 5' - TGCGATCTCTGCCTATCGCC - 3' | 5' - TGGATTTAAGCGGACAGCCTGC - 3' |
| IFIT2 | 4A | 5' -TAGGACACGCTGTGGCTCATCT - 3' | 5' - GAGCATGGAGGCTGGCAAGAA - 3' |
| IFIT3 | 4A | 5' - AACTGGGCCGCCTGCTAAGG - 3' | 5' - TCTGGGACTGGAGCTGACTGC - 3' |
| OAS1 | 4A | 5' - GCCTCATCCGCCTAGTCAAGC - 3' | 5' - CATGCTCCCTCGCTCCCAAG - 3' |
| OAS2 | 4A | 5' - GCTCCCGGCCCACCAAACTA - 3' | 5' - GACCCCTTTGGCTTCAGTTTCCT - 3' |
| OAS3 | 4A | 5' - AGCTGGTCACCCAGTACCGC - 3' | 5' - GGATGATAGGCCTGGGCTTCTG - 3' |
| MX1 | 4A | 5' - CCGACACGAGTTCCACAAATGG | 5' - CCTGGCAGCTCTCTACCACGA - 3' |
| RSAD2 | 4A | 5' - TTGTGCTGCCCCTTGAGGAA - 3' | 5' - CCCAGGTATTCTCCCCGGTCT - 3' |
| B2M | 1C | 5' - ATGAGTATGCCTGCCGTGTG - 3' | 5' - CCAAATGCGGCATCTTCAAA - 3' |
| HuP0 | 4A | 5' - GCTTCCTGGAGGGTGTCC -3' | 5' - GGACTCGTTTGTACCCGTTG -3 |

**References**

1. Lachmandas, E., van den Heuvel, C.N., Damen, M.S., Cleophas, M.C., Netea, M.G., and van Crevel, R. 2016. Diabetes Mellitus and Increased Tuberculosis Susceptibility: The Role of Short-Chain Fatty Acids. *J Diabetes Res* 2016:6014631.

2. Zhu, C.T., and Rand, D.M. 2012. A hydrazine coupled cycling assay validates the decrease in redox ratio under starvation in Drosophila. *PLoS One* 7:e47584.

3. Dobin, A., Davis, C.A., Schlesinger, F., Drenkow, J., Zaleski, C., Jha, S., Batut, P., Chaisson, M., and Gingeras, T.R. 2013. STAR: ultrafast universal RNA-seq aligner. *Bioinformatics* 29:15-21.

4. Anders, S., Pyl, P.T., and Huber, W. 2015. HTSeq--a Python framework to work with high-throughput sequencing data. *Bioinformatics* 31:166-169.

5. Love, M.I., Huber, W., and Anders, S. 2014. Moderated estimation of fold change and dispersion for RNA-seq data with DESeq2. *Genome Biol* 15:550.

6. Varemo, L., Nielsen, J., and Nookaew, I. 2013. Enriching the gene set analysis of genome-wide data by incorporating directionality of gene expression and combining statistical hypotheses and methods. *Nucleic Acids Res* 41:4378-4391.

7. Liberzon, A., Birger, C., Thorvaldsdottir, H., Ghandi, M., Mesirov, J.P., and Tamayo, P. 2015. The Molecular Signatures Database (MSigDB) hallmark gene set collection. *Cell Syst* 1:417-425.

8. Becher, B., Schlitzer, A., Chen, J., Mair, F., Sumatoh, H.R., Teng, K.W., Low, D., Ruedl, C., Riccardi-Castagnoli, P., Poidinger, M., et al. 2014. High-dimensional analysis of the murine myeloid cell system. *Nat Immunol* 15:1181-1189.

9. Bandura, D.R., Baranov, V.I., Ornatsky, O.I., Antonov, A., Kinach, R., Lou, X., Pavlov, S., Vorobiev, S., Dick, J.E., and Tanner, S.D. 2009. Mass cytometry: technique for real time single cell multitarget immunoassay based on inductively coupled plasma time-of-flight mass spectrometry. *Anal Chem* 81:6813-6822.

10. Levine, J.H., Simonds, E.F., Bendall, S.C., Davis, K.L., Amir el, A.D., Tadmor, M.D., Litvin, O., Fienberg, H.G., Jager, A., Zunder, E.R., et al. 2015. Data-Driven Phenotypic Dissection of AML Reveals Progenitor-like Cells that Correlate with Prognosis. *Cell* 162:184-197.

11. Chen, H., Lau, M.C., Wong, M.T., Newell, E.W., Poidinger, M., and Chen, J. 2016. Cytofkit: A Bioconductor Package for an Integrated Mass Cytometry Data Analysis Pipeline. *PLoS Comput Biol* 12:e1005112.
